# Genome-wide identification, characterization, and expression analysis of the sweet potato (*Ipomoea batatas* [L.] Lam.) *ARF*, *Aux/IAA*, *GH3,* and *SAUR* gene families

**DOI:** 10.1186/s12870-023-04598-w

**Published:** 2023-12-07

**Authors:** Sarah R. Mathura, Fedora Sutton, Valerie Bowrin

**Affiliations:** 1 The Department of Life Sciences, The University of the West Indies, St. Augustine, Trinidad & Tobago; 2 ScienceVisions Inc, Brookings, SD USA

**Keywords:** ARF, Aux/IAA, GH3, SAUR, Sweet potato, Tuberization

## Abstract

**Background:**

Auxins are known to have roles in the tuberization process in sweet potato (*Ipomoea batatas* [L.] Lam.) and these effects are mediated by various auxin signalling gene families. In this study, an analysis of the sweet potato genome was performed to identify the *ARF*, *Aux/IAA*, *GH3*, and *SAUR* auxin signalling gene family members in this crop.

**Results:**

A total of 29 ARF, 39 Aux/IAA, 13 GH3, and 200 SAUR sequences were obtained, and their biochemical properties and gene expression profiles were analysed. The sequences were relatively conserved based on exon–intron structure, motif analysis, and phylogenetic tree construction. In silico expression analyses of the genes in fibrous and storage roots indicated that many sequences were not differentially expressed in tuberizing and non-tuberizing roots. However, some *ARF*, *Aux/IAA*, and *SAUR* genes were up-regulated in tuberizing storage roots compared to non-tuberizing fibrous roots while many *GH3* genes were down-regulated. Additionally, these genes were expressed in a variety of plant parts, with some genes being highly expressed in shoots, leaves, and stems while others had higher expression in the roots. Some of these genes are up-regulated during the plant’s response to various hormone treatments and abiotic stresses. Quantitative RT-PCR confirmation of gene expression was also conducted, and the results were concordant with the in silico analyses. A protein–protein interaction network was predicted for the differentially expressed genes, suggesting that these genes likely form part of a complex regulatory network that controls tuberization. These results confirm those of existing studies that show that auxin signalling genes have numerous roles in sweet potato growth and development.

**Conclusion:**

This study provides useful information on the auxin signalling gene families in *Ipomoea batatas* and suggests putative candidates for further studies on the role of auxin signalling in tuberization and plant development.

**Supplementary Information:**

The online version contains supplementary material available at 10.1186/s12870-023-04598-w.

## Background

Auxin is an important plant phytohormone that is involved in a variety of processes which include: apical dominance, vascular tissue differentiation, lateral root elongation, fruit development, flowering, and stress responses [1–3]. Auxins exert their effects via signal transduction pathways which include many gene families such as auxin response factor (ARF), auxin/ indole-3-acetic acid (Aux/IAA), Gretchen-Hagen 3 (GH3), and Small Auxin-Up RNA (SAUR) [4].

ARFs are transcription factors that exert their effect by binding to auxin response elements (AuxREs) located in the promoter regions upstream of auxin-responsive genes [5]. The basic structure of a typical ARF includes three conserved domains: a DNA-binding domain (DBD), an auxin responsive (aux_resp) region, and an Aux/IAA domain. These structures have been described in great detail [6, 7]. The conserved DBD is located near the N-terminus of the sequence and functions by recognizing AuxREs in promoter regions which allows the ARF to bind to the DNA sequence. The aux_resp region is a conserved region located in the middle of the sequence. This sequence sometimes has an amino acid composition bias that allows the ARF to function as a transcriptional activator or a repressor. Glutamine (Q)-rich middle regions are present in ARFs that are transcriptional activators, while serine (S)-rich, serine and glycine (SG)-rich, and serine and proline (SP)-rich middle regions are present in ARFs that are transcriptional repressors. The Aux/IAA domain is located at the ARF’s C-terminus, and it has a PB1 domain that is similar to that of Aux/IAA proteins which allows for dimerization between both proteins.

The Aux/IAA gene family in plants has been reviewed by Luo et al. [8]. The genes encode short-lived nuclear proteins, which inhibit ARFs by binding to them under low auxin concentrations. At higher auxin concentrations, Aux/IAA proteins bind to the transport inhibitor response 1/auxin signalling F-Box (TIR1/AFB) complex, causing the rapid ubiquitination and degradation of Aux/IAA and the subsequent release of ARFs, which can activate transcription. The *GH3* gene family is responsible for maintaining auxin balance, but it does not seem to have a conserved domain [9]. The GH3 protein is responsible for forming conjugates between amino acids and the hormones: auxin (IAA), jasmonic acid (JA), and salicylic acid (SA). These conjugates are not biologically active and are targeted for ubiquitin degradation. The *SAUR* gene family regulates plant development by acting as an effector of hormone signals, and its transcription can be rapidly induced with 2–5 min of auxin signalling [10].

Due to the importance of auxin signalling proteins in plant developmental responses, identification and functional characterization of such proteins in various plants have been conducted. The *ARF*, *Aux/IAA*, *GH3*, and *SAUR* gene families have been characterized in several economically important crops such as *Arabidopsis thaliana* [11], castor bean [9], cucumber [12], cotton [10] and potato [13]. To date, the repertoire of auxin early response proteins in hexaploid sweet potato has not been fully characterized, despite their importance in the sweet potato tuberization process [14]. It is necessary to characterize these proteins to further understand their roles in sweet potato tuber initiation and development.

Sweet potato (*Ipomoea batatas* [L.] Lam.) is a hexaploid staple crop that is ranked sixth in importance worldwide among the food crops produced [15]. Consequently, decades of research have been conducted to investigate how this crop tuberizes, in order to improve yields. However, analysis of this crop at the molecular level is not as easy as with other economically important crops since its complex genome makes it difficult to obtain a complete reference genome [16]. In other tuberizing crops, such as *Solanum tuberosum* [17, 18], *Ipomoea trifida* [19], and *Manihot esculenta* [20], several auxin-responsive genes are up-regulated during tuberization.

This study seeks to characterize and investigate the expression of sweet potato *IbARF*, *IbAux/IAA*, *IbGH3*, and *IbSAUR* genes during tuberization. Phylogenetic analysis, motif analysis, and promoter analyses were performed. The expression studies of these genes in public databases were analysed and confirmed with qRT-PCR. Our results represent the first genome-wide characterization of the *ARF, Aux/IAA, GH3,* and *SAUR* genes in the hexaploid sweet potato. These results will facilitate better annotation of the sweet potato genome and provide insights on controlling the tuberization process, towards increasing crop production and food sustainability.

## Results

### Identification and characterization of *IbARF*, *IbIAA*, *IbGH3*, and *IbSAUR* genes

After HMMER searching, manual inspection of the domain organization via the NCBI CDD, removal of redundant sequences, and clustering of highly similar sequences, 29 *IbARF* sequences (*IbARF1* – *IbARF28*), 39 *IbAux/IAA* sequences (*IbIAA1* – *IbIAA33*), 13 *GH3* sequences (*IbGH3.1 – IbGH3.13*), and 200 *IbSAUR* sequences (*IbSAUR1 – IbSAUR200*) were obtained. Their predicted biochemical characteristics are summarized in Table 1 and Table S2. The predicted novel isoforms of the genes are listed in Table S6 (Additional File 16).

**Table 1:** Summary of *ARF*, *Aux/IAA* and *GH3* gene families in sweet potato

Name	Gene ID	Chromosome Location	Chromosome Position	Strand	CDS length	Protein Length (a.a.)	MW (kDa)	pI	Subcellular Location	No. of exons	Domain
IbARF1	g59391	LG14	28394145–28400481	+	2457	818	91.36	6.13	nucleus	15	DBD, ARF, CTD
IbARF2	g8727	LG2	33246806–33251837	+	2079	692	75.91	6.46	nucleus	3	DBD, ARF
IbARF3	g34371	LG9	2235070–2241480	-	2109	702	79.04	6.29	nucleus	15	DBD, ARF, CTD
IbARF4	g4753	LG2	3852087–3857844	+	2529	842	92.89	7.07	nucleus	12	DBD, ARF, CTD
IbARF5	g41733	LG11	2388778–2397000	+	2769	922	102.15	5.17	nucleus	14	DBD, ARF, CTD
IbARF6	g37210	LG9	23717784–23728660	+	2982	993	109.64	6.44	nucleus	17	DBD, ARF, CTD
IbARF7	g60744	LG15	5404732–5414608	+	3102	1033	114.46	6.34	nucleus	17	DBD, ARF, CTD
IbARF8	g29590	LG7	31355826–31362577	-	2658	885	98.42	6.16	nucleus	15	DBD, ARF, CTD
IbARF9	g35069	LG9	7162131–7166778	-	1953	650	72.31	6.78	nucleus	15	DBD, ARF, CTD
IbARF10	g48029	LG12	8572118–8578621	+	2412	803	89.26	5.66	nucleus	12	DBD, ARF, CTD
IbARF11	g35353	LG9	9203658–9208674	+	2559	852	94.34	6.40	nucleus	14	DBD, ARF, CTD
IbARF12	g499	LG1	2741507–2747778	-	2445	814	90.66	5.84	nucleus	14	DBD, ARF
IbARF13	g47890	LG12	7475602–7480416	+	1992	663	72.89	6.42	nucleus	6	DBD, ARF
IbARF14	g59133	LG14	26832217–26837751	+	2205	734	80.66	6.44	nucleus	11	DBD, ARF
IbARF15	g34554	LG9	3395918–3400037	-	1824	607	66.50	6.50	nucleus	11	DBD, ARF
IbARF16a	g31604	LG8	8196879–8205281	+	2343	780	86.97	5.92	nucleus	14	DBD, ARF, CTD
IbARF16b	g31598	LG8	8143819–8152148	+	2187	728	80.62	8.57	nucleus	16	DBD, ARF, CTD
IbARF17	g32239	LG8	13517466–13524143	+	2268	755	83.52	5.38	nucleus	16	DBD, ARF, CTD
IbARF18	g54977	LG13	27141643–27145793	-	1920	639	70.99	6.49	nucleus	5	DBD, ARF, CTD
IbARF19	g47893	LG12	7535283–7542098	-	2763	920	102.27	8.33	nucleus	7	DBD, ARF
IbARF20	g48000	LG12	8299289–8306959	+	2550	849	94.72	7.25	nucleus	8	DBD, ARF
IbARF21	g55041	LG13	27543052–27548748	-	3285	1094	121.78	6.14	nucleus	16	DBD, ARF, CTD
IbARF22	g42441	LG11	7199686–7206641	-	3174	1057	116.39	5.98	nucleus	17	DBD, ARF, CTD
IbARF23	g19483	LG5	21129257–21141322	-	1797	598	67.12	6.09	nucleus	16	DBD, ARF, CTD
IbARF24	g52627	LG13	10559464–10564889	-	2538	845	94.74	6.78	nucleus	16	DBD, ARF, CTD
IbARF25	g52675	LG13	11072641–11078076	+	2628	875	97.90	7.19	nucleus	15	DBD, ARF, CTD
IbARF26	g4723	LG2	3675472–3679198	+	2172	723	79.90	6.83	nucleus	5	DBD, ARF
IbARF27	g59859	LG15	3884–8941	-	1650	549	60.40	8.25	nucleus	5	DBD, ARF
IbARF28	g60835	LG15	6072825–6076451	-	2106	701	77.53	6.25	nucleus	4	DBD, ARF
IbIAA1	g5344	LG2	7548551–7549530	+	582	193	21.74	7.58	nucleus	2	I, II, III, IV
IbIAA2	g13864	LG4	7640002–7641426	-	585	194	21.44	5.17	nucleus	3	I, II, III, IV
IbIAA3	g8426	LG2	31203957–31205084	+	570	189	21.05	5.01	nucleus	3	I, II, III, IV
IbIAA4	g13218	LG4	3046681–3049178	+	714	237	26.61	5.35	nucleus	4	I, II, III, IV
IbIAA5a	g51202	LG13	267777–271437	-	540	179	19.89	8.34	nucleus	3	I, II, III, IV
IbIAA5b	g51419	LG13	1936588–1940243	-	435	144	15.99	5.78	nucleus	3	I, II, III
IbIAA6a	g13174	LG4	2739813–2742554	-	522	173	26.68	8.16	nucleus	5	I, II, III, IV
IbIAA6b	g13217	LG4	3025484–3028187	-	738	245	26.82	8.46	nucleus	5	I, II, III, IV
IbIAA7	g9596	LG3	1184368–1187595	+	729	242	26.08	7.68	nucleus	5	I, II, III, IV
IbIAA8	g29122	LG7	28177690–28182077	-	1164	387	41.55	8.76	nucleus	5	I, II, III, IV
IbIAA9	g42000	LG11	3987793–3991826	-	1032	343	37.45	5.83	nucleus	6	I, II, III, IV
IbIAA10a	g4276	LG2	658186–659812	+	354	117	12.97	5.80	nucleus	1	IV
IbIAA10b	g62815	LG15	21458790–21461191	+	1341	446	49.63	6.44	nucleus	5	IV
IbIAA11	g9720	LG3	1981869–1983222	+	462	153	17.56	4.75	nucleus	5	IV
IbIAA12	g8241	LG2	29885957–29888887	-	828	275	29.32	6.85	nucleus	5	I, II, III, IV
IbIAA13	g13071	LG4	2091248–2097890	-	798	265	29.04	9.42	nucleus	5	I, II, III, IV
IbIAA14	g8424	LG2	31190293–31193241	-	714	237	26.41	6.35	nucleus	4	I, II, III, IV
IbIAA15a	g51198	LG13	232831–238007	-	621	206	23.00	9.31	nucleus	5	I, II, III, IV
IbIAA15b	g51416	LG13	1910063–1911483	-	621	206	23.05	9.03	nucleus	5	I, II, III, IV
IbIAA16	g56225	LG14	5951428–5956751	-	681	226	24.38	7.64	nucleus	5	I, II, III, IV
IbIAA17	g13867	LG4	7684447–7687687	+	711	236	26.01	7.59	nucleus	5	I, II, III, IV
IbIAA18	g31376	LG8	6690272–6692986	-	960	319	35.31	9.04	nucleus	5	I, II, III, IV
IbIAA19	g9605	LG3	1275466–1278690	+	834	277	29.92	6.97	nucleus	6	I, II, III, IV
IbIAA20a	g31897	LG8	10676014–10678517	-	597	198	22.58	5.44	nucleus	4	II, III, IV
IbIAA20b	g62057	LG15	15608986–15612217	+	594	197	22.58	5.59	nucleus	4	II, III, IV
IbIAA20c	g62205	LG15	16828836–16831596	+	594	197	22.49	5.32	nucleus	4	II, III, IV
IbIAA21	g55517	LG14	1049110–1050985	-	1185	394	44.53	9.14	nucleus	5	III, IV
IbIAA22	g39034	LG10	6134522–6138092	-	1146	381	41.29	6.28	nucleus	7	I, II, III, IV
IbIAA23	g21833	LG6	7312394–7314366	+	429	142	16.01	5.02	nucleus	3	II, III
IbIAA24	g19769	LG5	23440642–23443051	+	975	324	35.54	8.09	nucleus	8	I, II, III, IV
IbIAA25	g55269	LG13	28900889–28902722	-	762	253	27.78	5.32	nucleus	3	I, II, III
IbIAA26	g61025	LG15	7370833–7373179	-	606	201	21.87	5.32	nucleus	4	II, III, IV
IbIAA27	g55286	LG13	29005161–29007909	-	1077	358	38.26	4.89	nucleus	5	I, II, III, IV
IbIAA28	g21443	LG6	4380350–4382045	+	762	253	27.84	6.16	nucleus	4	I, II, III, IV
IbIAA29	g21732	LG6	6736576–6739075	-	585	194	22.33	6.44	nucleus	4	III
IbIAA30	g21733	LG6	6746443–6748745	-	732	243	28.24	8.25	nucleus	5	II, III
IbIAA31	g36618	LG9	18867423–18871633	+	618	205	23.50	5.93	nucleus	4	II, IV
IbIAA32	g21837	LG6	7342737–7344682	-	666	221	25.35	8.42	nucleus	4	II, III, IV
IbIAA33	g16695	LG5	635787–636498	-	447	148	16.40	9.30	nucleus	2	III, IV
IbGH3.1	g59630	LG14	29651724–29654131	-	1797	598	66.44	5.43	plasma membrane, cytoplasm, nucleus	3	-
IbGH3.2	g29011	LG7	27375147–27377604	+	1800	599	66.92	5.95	nucleus	3	-
IbGH3.3	g28936	LG7	26815115–26817524	+	1311	436	48.89	9.03	nucleus	6	-
IbGH3.4	g30915	LG8	3596734–3599918	+	1671	556	63.18	5.78	plasma membrane, cytoplasm	4	-
IbGH3.5	g54101	LG13	21665190–21667469	-	1815	604	68.20	6.62	nucleus	3	-
IbGH3.6	g61115	LG15	7968829–7971680	+	1689	562	63.31	5.61	cytoplasm, nucleus	6	-
IbGH3.7	g30914	LG8	3588634–3590995	+	1803	600	67.59	5.85	plasma membrane, nucleus	3	-
IbGH3.8	g30916	LG8	3600274–3605749	+	2064	687	77.46	5.59	plasma membrane	4	-
IbGH3.9	g49834	LG12	21853291–21859368	+	1848	615	69.04	5.74	plasma membrane, nucleus	4	-
IbGH3.10	g54418	LG13	23757464–23761208	-	1725	574	65.15	6.25	cytoplasm, nucleus	5	-
IbGH3.11	g9512	LG3	515790–518455	-	1422	473	52.71	5.21	cytoplasm, chloroplast	4	-
IbGH3.12	g56407	LG14	7163225–7172836	+	1962	653	72.88	7.45	plasma membrane	7	-
IbGH3.13	g56453	LG14	7568229–7572565	-	1893	630	70.46	6.59	plasma membrane, cytoplasm	6	-

**Fig. 1:**
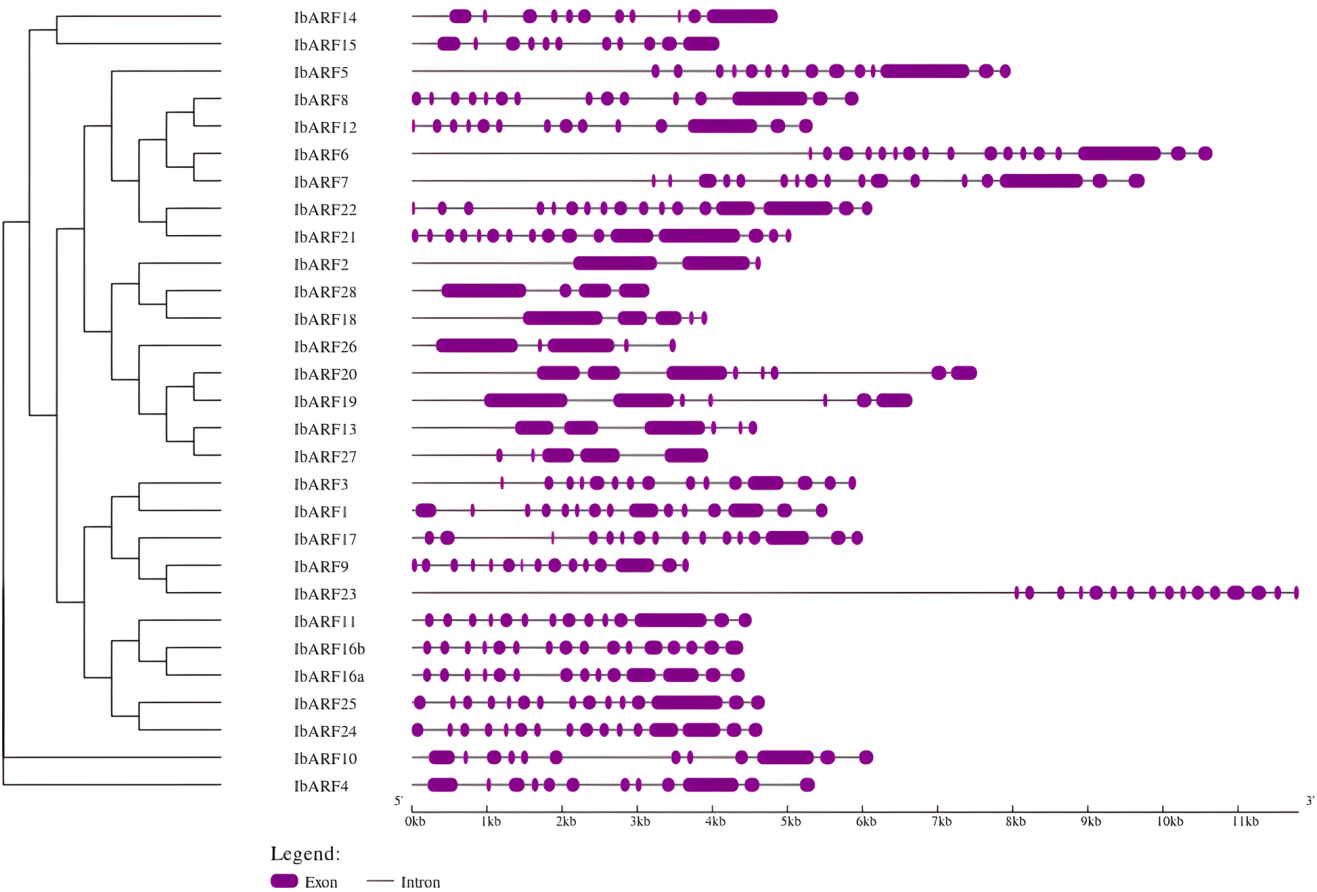
Map showing the intron–exon structure of the *IbARF* coding sequences (figure created on the GSDS server). The left panel illustrates a neighbour-joining (NJ) phylogenetic tree based on the aligned sequences with 1000 bootstrap replicates. Sequences with similar intron–exon structure cluster together in the NJ tree

The *IbARF*, *IbIAA*, *IbGH3*, and *IbSAUR* genes were distributed unevenly across the chromosomes, with the majority of the *IbSAURs* located on Chromosome 14. The genes with similar intron–exon arrangements clustered together on a phylogenetic tree (Figs. 1, S1, S2, S3). The proteins encoded by these genes showed a wide range of molecular weights (MWs) and predicted isoelectric points (pIs). Analysis of the domain organization in the NCBI CDD indicated that 19 of the ARF proteins had the canonical structure consisting of the B3 DNA-binding domain, the conserved middle region, and the conserved C-terminus domain. CDD analysis indicated that not all the IbIAA proteins were complete matches to the canonical Aux_IAA domain, with 16 sequences being truncated.

### Motif analysis of IbARF, IbIAA, IbGH3, and IbSAUR sequences

The results of investigation of the domain architecture of the protein sequences revealed the presence of several highly conserved motifs, many of which were functional domains that were present in the Interpro database.

*ARF* Of the 20 motifs searched, Motifs 1 and 5 represented the B3 DNA binding domain, as shown in Fig. S4. One or both of these motifs were found in all IbARF sequences. Motif 2 represented the aux_resp middle domain and this motif was observed in all the sequences. Motif 3 represented the PB1 domain (domains III and IV of Aux/IAA proteins). This motif was present in 22 proteins. Motifs 4, 11, 12, and 15 matched the ARF protein Interpro domain. Motif 15, which is rich in Q residues, was observed in the middle region of 7 proteins (ARF5, ARF6, ARF7, ARF8, ARF12, ARF21, and ARF22).

**Fig. 2:**
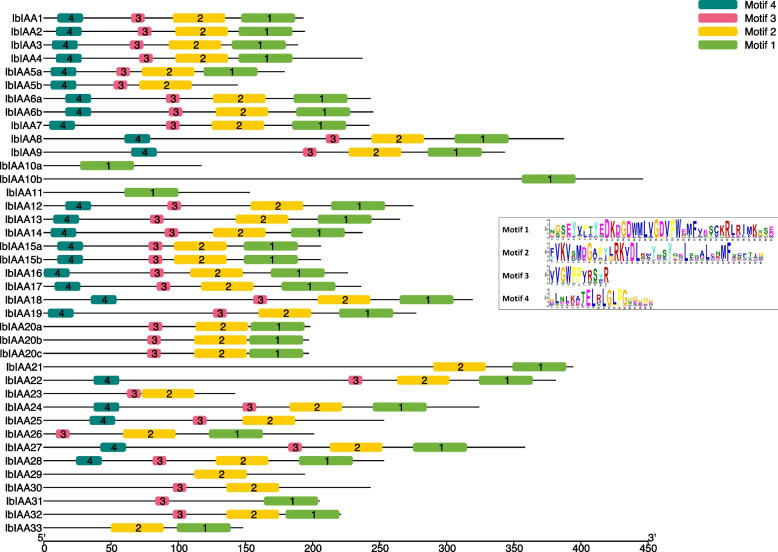
Conserved motifs in IbIAA sequences identified by the MEME software (Figure created using TBTools). There are no duplicated motifs within a sequence, and the order of motifs in the sequences is the same

*Aux/IAA* Of the four domains (I, II, III, and IV) found in canonical Aux/IAA proteins (Fig. 2, Table 1), four motifs were observed, each corresponding to one of the domains. Motif 4 was found in 25 sequences, corresponding to Domain I which contains the “LxLxL” ethylene response factor (ERF)-associated amphiphilic repression (EAR) motif [8]. Motif 3, which corresponds to Domain II, was present in 33 sequences and this motif contains the “GWPPV” degron sequence which controls the turnover of these sequences [8]. Motifs 2 and 1 corresponded to Domains III and IV respectively and both domains represent the Phox and Bem1p (PB1) domains (IPR000270) which allow Aux/IAA proteins to form homodimers with themselves or heterodimers with ARF proteins [8]. Motifs 2 and 1 were each found in 36 and 35 proteins, respectively. Twenty-three of the 39 IbIAA sequences had motifs that corresponded to all four domains (Motifs 1, 2, 3, 4) as seen in canonical Aux/IAA proteins.

*GH3* Motif analysis of the 13 GH3 protein sequences yielded 20 different motifs (Fig. S5). Motif 1 was found in all 13 sequences, and this motif corresponded to the GH3 family (IPR004993) in the Interpro database. All 13 sequences had a combination of Motifs 2–12 which also belonged to the GH3 family.

*SAUR* A maximum of ten motifs were observed upon examination of the SAUR protein sequences with MEME (Fig. S6) Motif 1 was found in 186 sequences and this motif represented a conserved SAUR domain in the Interpro database. Motif II was found in 169 sequences, while Motif III (which corresponded to the SAUR domain, pfam02519, in the CDD) was found in 63 sequences. Fourteen remaining sequences lacked Motif I.

### Phylogenetic analysis of IbARF, IbIAA, IbGH3, and IbSAUR sequences

**Fig. 3:**
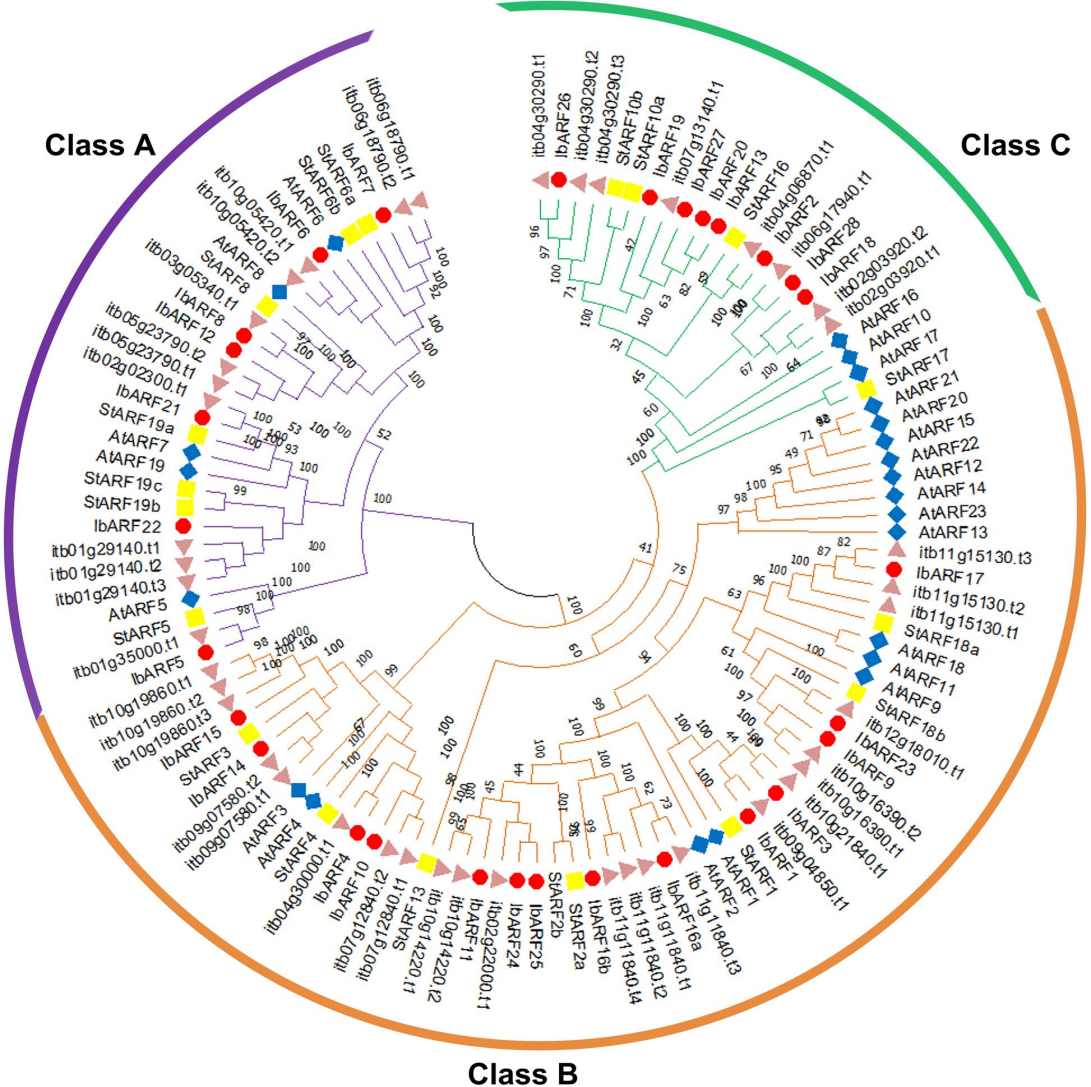
Neighbour-joining phylogenetic tree showing the phylogenetic relationships between ARF sequences. The trees were constructed using 1000 bootstrap replicates in MEGA 11. The *I. batatas* sequences were represented by a red circle. The numbers on each branch represent the percentage of replicate trees that clustered together in the bootstrap test. The branches are coloured into three classes according to the classification of Finet et al. [22] and used by Song et al. [13]

The Neighbour-joining phylogenetic tree (Fig. 3) displays the grouping of the ARF proteins into three distinct classes, A, B, and C. Class A contains 7 IbARFs with Q-rich middle regions. Class B and Class C contain 14, and 8 IbARFs, respectively. Classes B and C are rich in serine, proline, and threonine [21].

The IbIAA phylogenetic tree (Fig. S7) shows that the sequences are grouped into 6 clades which we labelled Clades A-E (as previously described by Wu et al. [23]) and Clade F, which contains non-canonical IAA sequences that were excluded from the study mentioned above. All the IbIAA proteins were distributed among all 6 clades.

The GH3 phylogenetic tree is illustrated in Fig. S7. The sequences are clustered into 3 groups as previously reported [24], with the IbGH3 sequences present in only two groups. There were no IbGH3 sequences in Group 3, which consisted of only AtGH3 proteins.

Fig. S7 illustrates the phylogenetic tree created from 79 *A. thaliana* sequences, 199 IbSAUR sequences (IbSAUR151 excluded), and 58 *Oryza sativa* protein sequences. The sequences were grouped into clades that were described by Zhang et al. [25]. Clade I and Clade V had the largest number of IbSAUR members, possibly arising from gene duplication events. All ten clades had members of the IbSAUR family. None of the DEGs belonged to Clade I, but *IbSAUR33* and *IbSAUR62* were part of Clade V.

### In silico analysis of *cis*-acting regulatory elements (CREs)

PlantCARE analysis of the 2,000 bp region upstream of the start codon for each of the genes identified revealed a variety of core promoter elements (Table S4). All the *ARF*, *IAA*, and *GH3* genes had light-responsive elements in their promoter sequences, with most genes having multiple types of light-responsive elements. Light responsive elements were found in 196 of the *SAUR* genes.

Most of the promoter sequences contained at least one CRE involved in responsiveness to various hormones such as auxin, gibberellin (GA), SA, ethylene, abscisic acid (ABA), and JA. Four types of auxin-responsive elements were found in the *ARF*, *Aux/IAA*, and *GH3* promoter sequences: AuxRR-core, TGA-box, AuxRE, and TGA-element. Thirty-nine *SAUR* genes had auxin-responsive elements. These results are consistent with those of Feng et al. [9], who found that not all *ARF*, *Aux/IAA*, and *GH3* promoter sequences from castor bean (*Ricinus communis*) had auxin-responsive elements.

Some of the promoter sequences had elements associated with responding to abiotic and biotic stresses such as low temperature (LTR), abiotic stress (as-1), wounding (WUN-motif, WRE3), drought (MBS), and low oxygen (GC-motif). Some sequences had *cis*-elements that are involved in plant development processes such as meristem expression (CAT-box), circadian control (circadian), seed-specific regulation (RY-element), endosperm expression (GCN4-motif), and negative regulation of phloem expression (AC-I, AC-II). Some promoter sequences had binding site-related elements such as AT-rich element, Myb-binding site, Box III, and Unnamed_1 (Table S4). A more detailed breakdown of the numbers of the different types of CREs found in the DEGs observed during tuber initiation is presented in Table S5.

### In silico gene expression analysis of auxin signalling genes

**Fig. 4:**
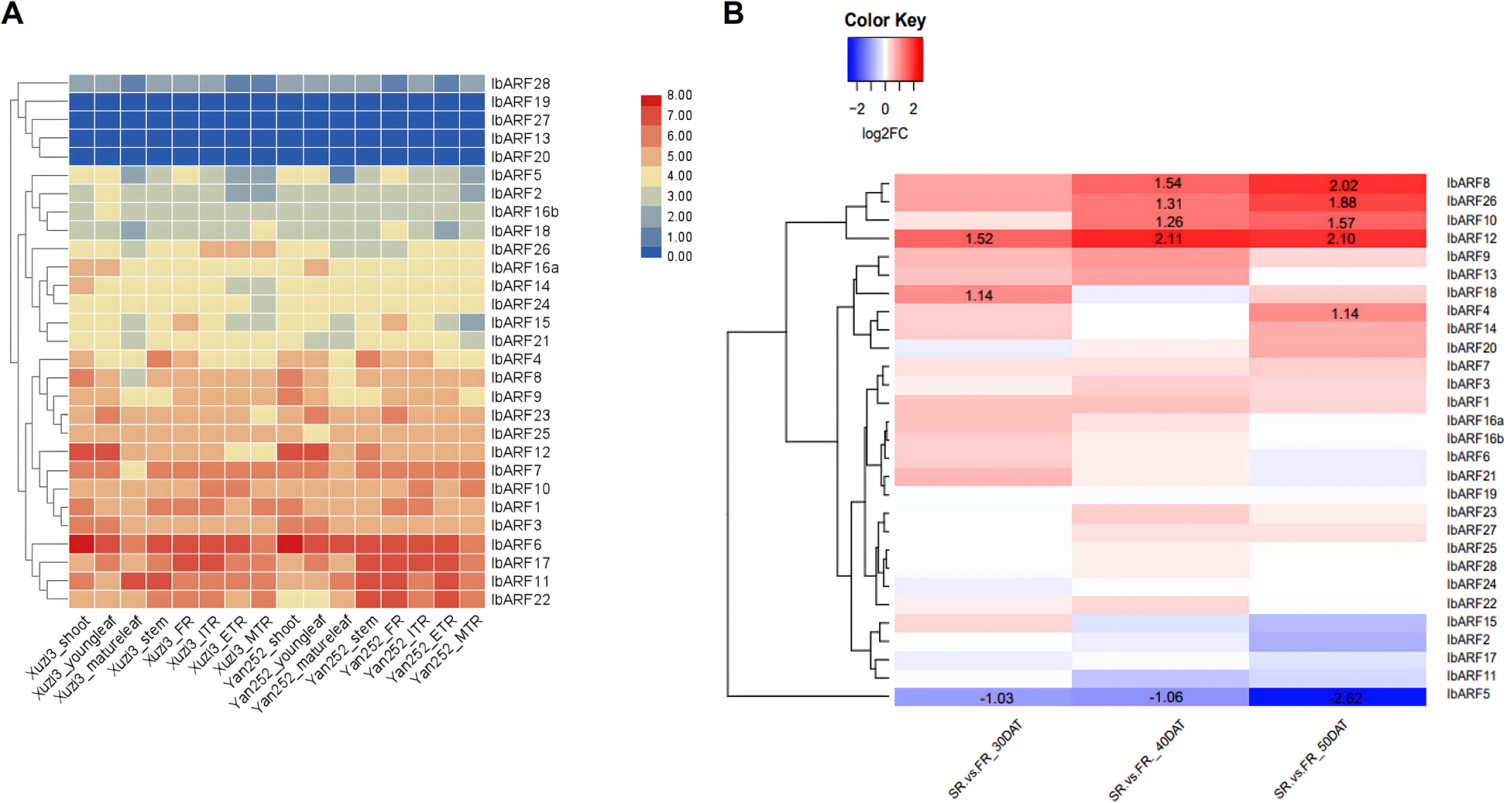
Heatmap showing the gene expression of *IbARF* genes obtained from: **a** RNA-seq data [26] obtained from various tissues for both Xuzi3 and Yan252 sweet potato cultivars. The colour scale bar represents the log_2_(FPKM + 1) values. **b** RNA-seq data [16] obtained from FRs and SRs at various stages of development. The colour scale bar shows that blue indicates down-regulated expression and red represents up-regulated expression. Colours represent log_2_FC. The raw log_2_FC data is indicated for statistically significant (adj. *p*-val. < 0.05) differential expression only

**Fig. 5:**
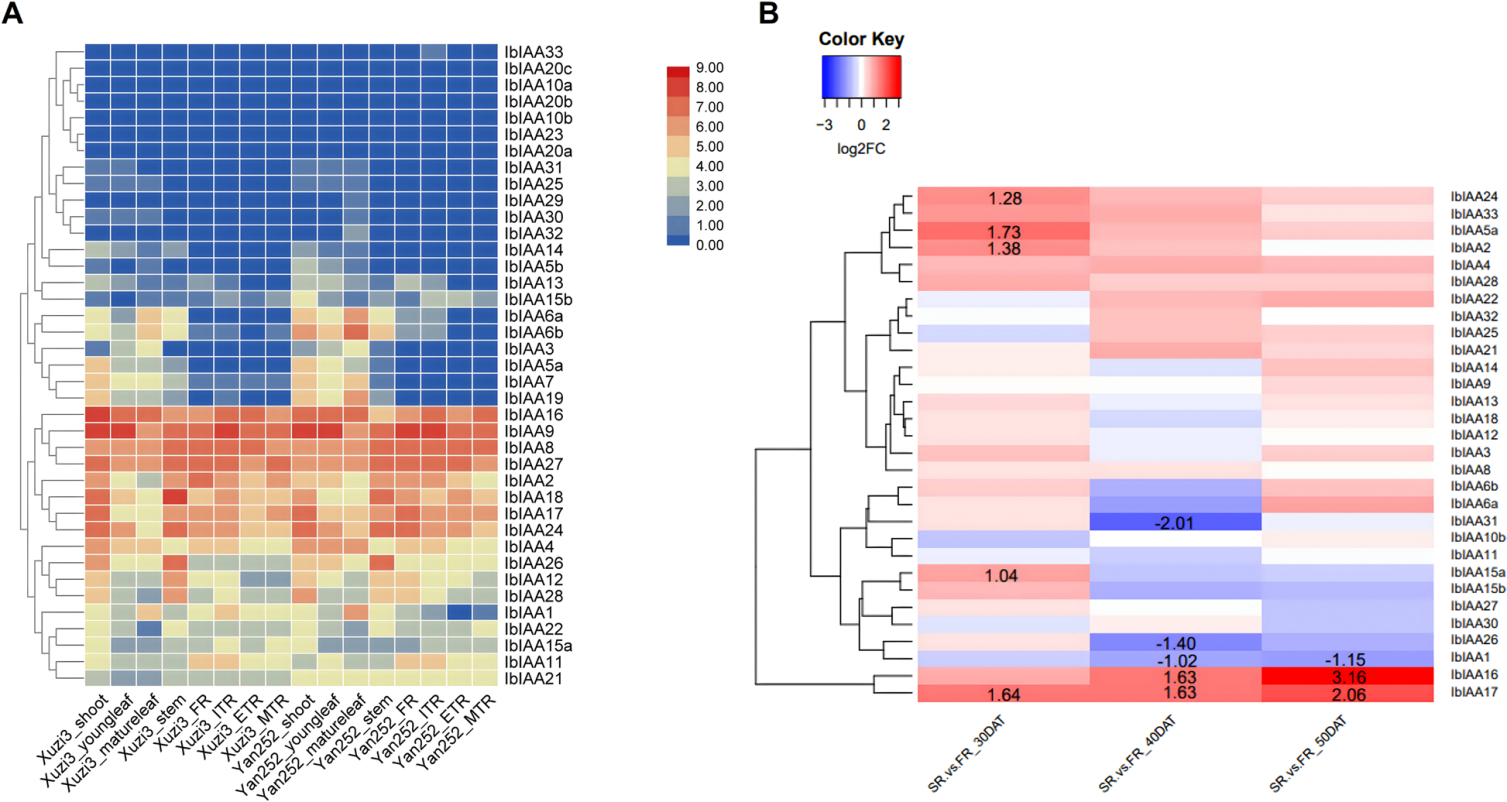
Heatmap showing the gene expression of *IbIAA* genes obtained from: **a** RNA-seq data [26] obtained from various tissues for both Xuzi3 and Yan252 sweet potato cultivars. The colour scale bar represents the log_2_(FPKM + 1) values. **b** RNA-seq data [16] obtained from FRs and SRs at various stages of development. The colour scale bar shows that blue indicates down-regulated expression and red represents up-regulated expression. Colours represent log_2_FC. The raw log_2_FC data is indicated for statistically significant (adj. *p*-val. < 0.05) differential expression only

**Fig. 6:**
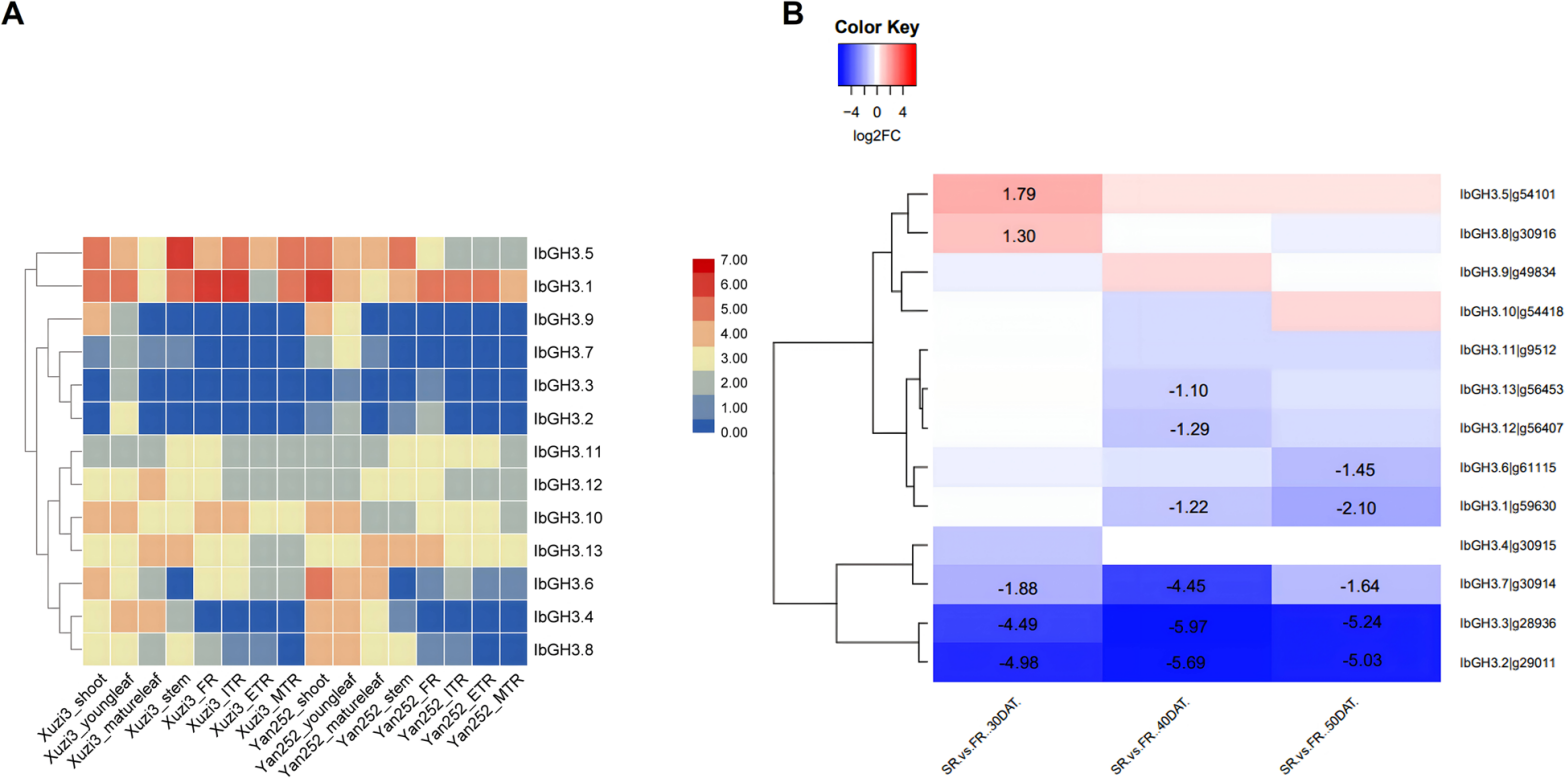
Heatmap showing the gene expression of *IbGH3* genes obtained from: **a** RNA-seq data [26] obtained from various tissues for both Xuzi3 and Yan252 sweet potato cultivars. The colour scale bar represents the log_2_(FPKM + 1) values. **b** RNA-seq data [16] obtained from FRs and SRs at various stages of development. The colour scale bar shows that blue indicates down-regulated expression and red represents up-regulated expression. Colours represent log_2_FC. The raw log_2_FC data is indicated for statistically significant (adj. *p*-val. < 0.05) differential expression only

The results of in silico gene expression analyses of each auxin signalling gene are presented (Figs. 4, 5 and 6 and Figs. S8, S9 and S10).

*ARF* All 29 *ARF* genes were expressed across all the plant tissues studied (Fig. 4a). However, some genes (*IbARF13*, *IbARF19*, *IbARF20*, and *IbARF27*) had FPKM (fragments per kilobase per million) values that were less than one and were only expressed in non-root plant parts. The gene expression in both cultivars was generally similar, although some genes, such as *IbARF22* (which has higher expression in *Yan252* root tissues), have cultivar-specific expression. *IbARF3* and *IbARF12* had very high expression in the shoots and young leaves. *IbARF4* had very high expression in the stems of both cultivars. *IbARF17* had high expression in roots and green stems.

Since FPKM should not be used to make statistical comparisons across samples, a separate dataset was used to calculate fold changes based on DESeq2 normalization (Fig. 4b). No DEGs were obtained for the 20 DAT vs. 10 DAT comparison and this comparison was not investigated further. The expression of *ARF8*, *ARF10*, *ARF12*, and *ARF26* genes were significantly higher in storage roots (SRs) compared to fibrous roots (FRs) from 40 DAT and beyond. *ARF18* was only significantly differentially expressed at the 30 DAT stage only. *ARF4* was significantly up-regulated in SRs at the 50 DAT stage. Many of the *ARF* sequences were not significantly differentially expressed at any of the stages that were investigated. *IbARF5* was significantly down-regulated in SRs compared to FRs. Most of the *IbARFs* had no significant change in expression or showed down-regulation in response to ABA, MeJA, SA, drought, salt, or cold (Fig. S9). The only exceptions were *IbARF18* and *IbARF23*, which were up-regulated in leaves in response to cold (Fig. S9).

*IAA* The expression of the *IAA* genes is summarized in Fig. 5. IbIAA17 was strongly expressed in shoots, FRs, and initiating tuberous root (ITRs). As shown in Fig. 5a, some genes had the highest FPKM values in shoots, leaves, and stems, while other genes, such as *IbIAA27* had high expression in all the tissues. *IbIAA16* had high FPKM values in expanding tuberous roots (ETRs), ITRs, shoots, and young leaves. There were several genes (*IbIAA5a*, *IbIAA6*) that had negligible expression in roots but were expressed in the other plant parts. Figure 5b illustrates the fold changes observed between SR and FR. Some genes were up-regulated at the 30 DAT time point only (*IbIAA*-*2*, -*5a*, -*15*, -*24*) while *IbIAA16* and *IbIAA17* were up-regulated at 40 DAT and 50 DAT. *IbIAA*-*1*, -*26*, and -*31* were down-regulated at 40 DAT while the other genes were not differentially expressed. Most of the *IbIAAs* had no significant change in expression or showed down-regulation in response to ABA, MeJA, SA, drought, salt, or cold (Fig. S9). *IbIAA-1*, *-2*, *-4*, *-11*, *-29*, *-30*, and *-32* were up-regulated in one or more plant part in response to cold treatment. *IbIAA-12* and *-26* were up-regulated in response to ABA, MeJA, drought, and salt treatments, while *IbIAA18* was up-regulated in response to drought in FRs only.

*GH3* The expression patterns of *GH3* genes are summarized in Fig. 6. All the *GH3* genes were expressed across the tissues that were investigated. Many of the *GH3* genes had their highest FPKM values in shoots and young leaves and the gene expression was similar across cultivars. Two genes (*IbGH3.1*, *IbGH3.10*) had high FPKM values in shoots, young leaves, FR and ITR. *IbGH3.11* had higher FPKM in stem and root tissue than in shoots and leaves (Fig. 6a). The results of differential expression of *GH3* genes between SR and FR, are illustrated in Fig. 6b. Seven of the 13 *GH3* genes were down-regulated at one or more time points, with *GH-3.2*, -*3.3*, and -*3.7* being significantly down-regulated at all three time points. These three genes belong to Group II of the phylogenetic tree (Fig. S7c). *GH3.5* and *GH3.8* were up-regulated at the 30 DAT stage only. *GH.12* and *GH3.13* were down-regulated at 40 DAT only and these were the only DEGs from the *GH3* gene family that belonged to Group I of the phylogenetic tree. Of the 13 *IbGH3* genes, only five showed up-regulated gene expression in response to one or more of the hormone or stress treatments in Fig. S9. *IbGH3.1* was up-regulated in response to cold, while *IbGH3.5* and *IbGH3.11* were up-regulated in response to SA and MeJA, respectively (Fig. S9). *IbGH3.2* and *IbGH3.3* showed up-regulation under MeJA, drought, and cold treatments.

*SAUR* The expression of the *SAUR* genes is summarized in Fig. S8. Of the 200 *SAUR* genes identified, 8 (*IbSAUR-92*, -*107*, -*115*, -*132*, -*133*, -*155*, -*163,* -*190*) were not expressed across any of the tissue types for either cultivar. As shown in Fig. S8a, the tissue-specific expression was similar for both cultivars, with many genes having the highest FPKM values being observed mostly in the shoots and mature leaves and lower expression in roots. Some genes (such as *IbSAUR*-*10, -52*, -*60*) had highest expression in shoots and young leaves. More than 50% of the *IbSAUR* genes were not expressed in any of the root tissue types. There were some variations in gene expression between cultivars, such as for *IbSAUR35* which was higher expressed in Yan252 roots than in Xuzi3 roots. Fig. S8b shows the fold-changes between SR and FR and several SAURs that were only expressed at certain time points. Some genes (*IbSAUR-2*, -*3*, -*12*, -*13*, -*47*, -*48*, -*49*, -*61*, -*64*) were significantly up-regulated (log_2_FC > 2) in SR vs. FR across one or more time points, while others (*IbSAUR*-*9*, *10*, -*34*, -*62*) were down-regulated. *IbSAUR32* was highly expressed across all tissues in both cultivars, but it was not differentially expressed between SR and FR. Most of the *IbSAUR* genes did not show differential expression, or were down-regulated in response to ABA, MeJA, SA, drought, salt, or cold treatments (Fig. S10). *IbSAUR8* was up-regulated in response to SA and drought, while *IbSAUR9* was up-regulated in response to drought and salt. The remaining up-regulated *IbSAURs* were up-regulated in response to ABA (*IbSAUR48* and *IbSAUR61*), MeJA (*IbSAUR-29*, *-31*, *-63*, and *-168*), SA (*IbSAUR1* and *IbSAUR65*), and cold (*IbSAUR-37*, *-71*, *-98*, and *-118*).

### qRT-PCR confirmation of expression analysis

In order to validate the expression observed in the in silico gene expression analysis, qRT-PCR experiments were conducted using tissue from 5 plant parts (GS—green stem, PR—pencil root, SR—storage root, FR—fibrous root, L—leaf). PCR efficiencies ranged from 1.85 to 2.02 (except for IbARF7 which had an efficiency of 1.8).

**Fig. 7:**
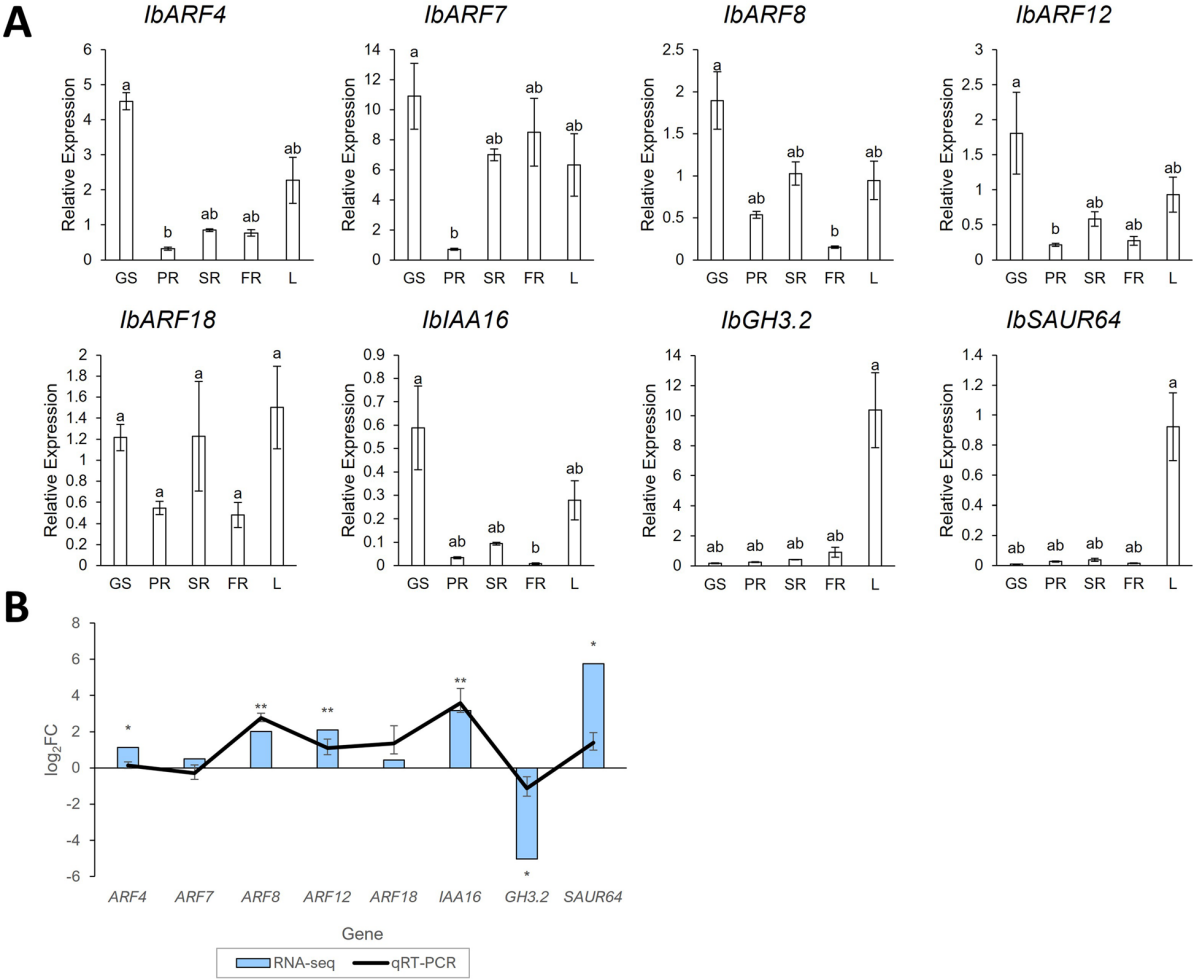
qRT-PCR confirmation of in silico gene expression. **a** qRT-PCR results showing expression of genes taken from RNA sampled from 3 pooled biological replicates of different sweet potato plant parts. The bars represent the mean ± standard error (SE) (*n* = 3). GS: green stem; PR: pencil root; SR: storage root; FR: fibrous root; L: leaf. Bars not sharing a common letter showed significant differences in gene expression using the Kruskal–Wallis H test (*p* < 0.05) **b** Comparison of log_2_FC data for SR vs. FR from in silico and qRT-PCR gene expression data. Asterisks indicate statistically significant (*p*-value < 0.05) log_2_FC (found using DESeq2 for in silico data and Student’s *t*-test for qRT-PCR data). A single asterisk indicates statistically significant log_2_FC from the RNA-seq data only, while 2 asterisks indicate statistically significant log_2_FC for both the RNA-seq and qRT-PCR data. The error bars for the qRT-PCR data represent the confidence intervals derived from the mean ± SE of the ΔΔCT values (*n* = 3)

For most of the genes, the highest gene expression was observed in the stem and/or leaf, which was also observed in the in silico analysis (Fig. 7a). The expression of *IbARF4* and *IbARF7* were similar in SRs and FRs, while for the remaining genes (except *IbGH3.2*), their expression was twofold higher (or more) in the SR relative to the FR (Fig. 7b). *IbGH3.2* was twofold down-regulated in the SR vs. FR. Therefore, there is concordance between the expression observed in the public datasets and the results from this study. An interesting observation was that for all the genes that were investigated, the PR and FR expression values were similar, but this trend was not observed for *IbARF7*, where the PR expression was much lower than that of the SR and FR.

### Predicted PPI network of proteins encoded by DEGs

**Fig. 8:**
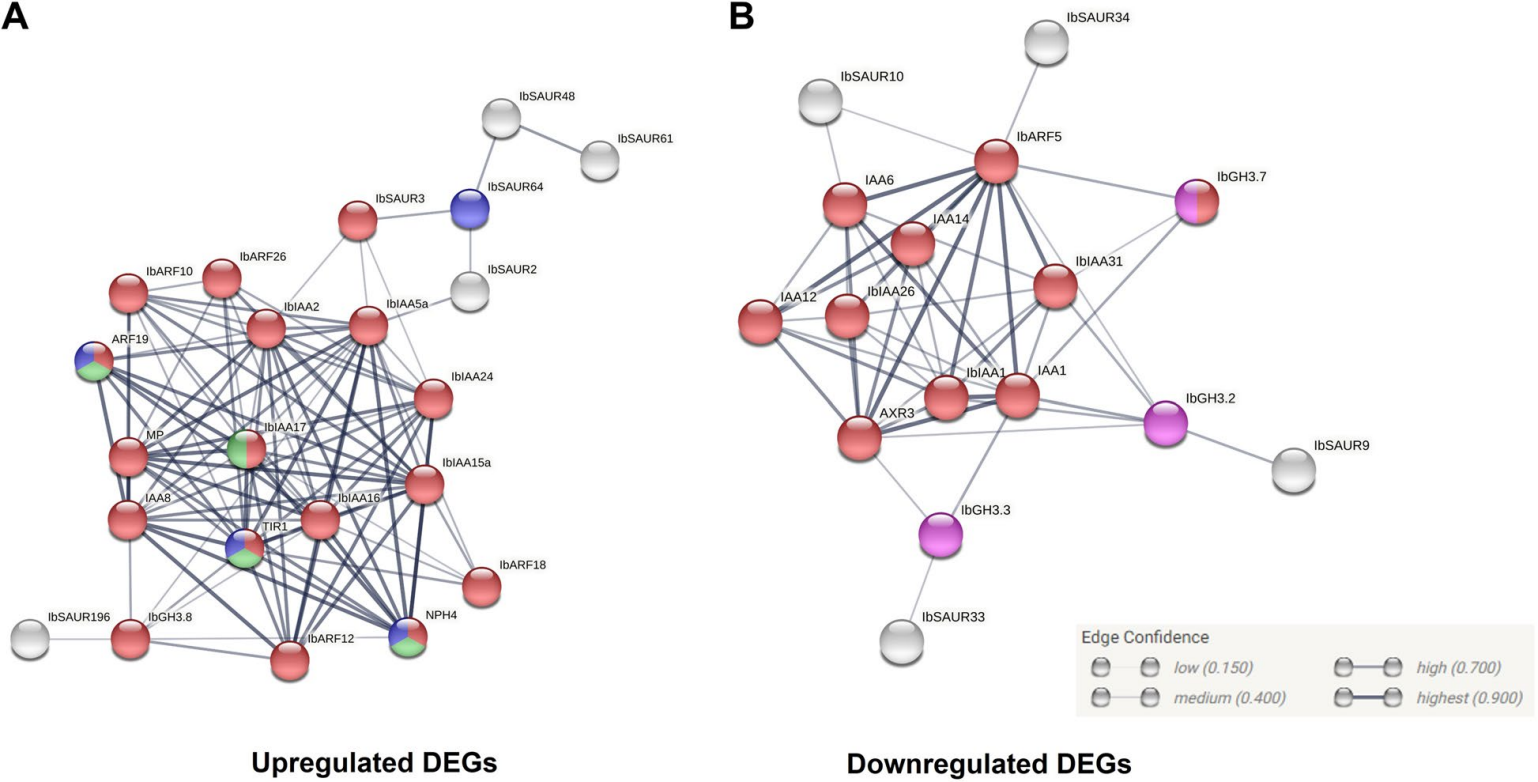
STRINGdb Protein–protein interaction diagram illustrating predicted interactions between the sweet potato auxin signalling genes based on their homology to *A. thaliana* proteins and their interactions. The nodes represent the auxin signalling proteins and the edges represent predicted interactions. No more than five interactors were shown in the first shell and zero interactors were shown for the second shell. Coloured nodes are those enriched with a Gene Ontology (GO) term with an FDR < 0.01—red: GO:0009734 (Auxin-activated signaling pathway); blue: GO:0009723 (Response to ethylene); green: GO:0010102 (Lateral root morphogenesis); purple: GO:0016881 (Acid-amino acid ligase activity). White nodes had no significant GO enrichment. The DEGs were prefixed with the species abbreviation, Ib **a** Up-regulated DEGs **b** Down-regulated DEGs. (inset) The thicknesses of the edges are proportional to the confidence of the prediction (only medium confidence score of 0.400 or higher shown)

A protein–protein interaction network for the proteins encoded by all DEGs was constructed based on the known interactions of *Arabidopsis* homologs (Fig. 8). This network was constructed to examine whether the different DEGs belonging to the same gene family had unique interaction partners, which could then be used to elucidate their roles in tuberization. The majority of the DEGs interacted with other proteins encoded by the DEGs, for example, the interactions with high confidence (score of 0.7 or more) occurred between ARF and IAA proteins (Fig. 8). Figure 8a shows that ARF5 (MONOPTEROS—MP), IbARF10 and IbARF12 were predicted to have high confidence interactions with multiple IAA proteins—IbARF10 and IbARF12 (or IbARF8) were predicted to interact with IbIAA-5a, -15a, -16 while IbARF12 was also predicted to interact with IbIAA-2, -17, -24. IbARF5 (or MP) was not only predicted to interact with all the aforementioned up-regulated IbIAA proteins but was also predicted to interact with the down-regulated IbIAA-1, -26, -31 (Fig. 8b). The IbGH3 and IbSAUR proteins were predicted to interact with other auxin signalling proteins, for example, GH3.2 was predicted to interact with IbARF5, IbIAA31 and IbSAUR9 while IbGH3.8 was predicted to interact with IbARF12, IbIAA16, and IbSAUR196. Except for MP, there was no overlap between the first shell interactors for the proteins of the up-regulated and down-regulated DEGs, for example, TIR1 was observed in the up-regulated network only, whereas several Aux/IAA proteins (IAA1, IAA6, AXR3) were observed in the down-regulated network.

## Discussion

### Characterization of sweet potato auxin signalling genes

In this study, we characterized the repertoire of the auxin regulated genes (*ARF*, *Aux/IAA*, *GH3*, and *SAUR*) in the hexaploid *I. batatas* genome. The 29 characterized *IbARF* genes are similar in number to that described in other crops [13, 19]. Our identification of 39 *IbAux/IAA* genes is also in agreement with that found in: *Populus trichocarpa*, tomato, and potato [18, 27, 28]. Likewise, the number of *IbGH3* genes are similar to that in other species, such as *Arabidopsis* [29], *Zea mays* [30], *O. sativa ssp. Japonica* [31], and *Solanum lycopersicum* [32]. This trend was not seen for the *SAUR* gene family, which had more *SAUR* genes than that of the other species in the phylogenetic tree (Fig. S7d).

Biochemical characterization of the auxin signalling protein sequences gave insight into their domain organization. Of interest, is that IbARF12 and IbARF26 do not have the canonical ARF domain structure, which indicates that non-canonical auxin signalling pathways may be involved during SR initiation. Additionally, if the ARF middle region is rich in glutamine (Q), serine (S), and leucine (L), it may function as an activator but if it is serine (S), proline (P), leucine (L), and glycine (G)-rich, then it may function as a repressor [13]. The up-regulated expressions of ARF8 and ARF12 give evidence for them being transcriptional activators based on their Q-rich domains. Some of the Aux/IAA sequences are truncated, such as IbIAA31, so further work is required to understand how they can modulate auxin responses. The high conservation of GH3 amino acid residues indicates that orthologous GH3 genes between *A. thaliana* and *I. batatas* have similar specificity for the amino acids they conjugate. Additionally, most of the SAUR proteins had the highly conserved SAUR domain (represented by Motif I) which is likely involved in the mechanism of action of these genes [33]. Sun et al. [10] reported a highly similar Motif I that was conserved across 7 plant species, so that Motif I is likely to be crucial for SAUR activity. The IbSAUR proteins that lack Motif I and are possibly pseudogenes.

Phylogenetic analysis also showed the evolutionary conservation of *I. batatas* auxin signalling sequences as they clustered together into distinct clades. The IbARF sequences clustered into 3 groups, which was consistent with the findings of Song et al. [13] and Zhang et al. [34]. All Class A *A. thaliana* ARFs are activators, and based on homology, the same can be said for Class A IbARFs. Members of Class B and Class C *A. thaliana* ARFs have been identified as transcriptional repressors [21] and based on homology, many of the Class B and Class C IbARFs may act as repressors.

The GH3 phylogenetic tree grouped the IbGH3 sequences by their homology to *A. thaliana* sequences. In *A. thaliana*, Group I GH3 proteins catalyse the conjugation of JA and amino acids [35] while Group II AtGH3 proteins conjugate auxin to amino acids [36]. It is possible that the corresponding IbGH3 members of the same group share the same function. The conjugation of IAA to amino acids by members of the GH3 family is very important to control the level of endogenous IAA. This is because auxin usually exerts its effects via the creation of local auxin gradients, in which the ratio of free auxin to auxin conjugates are kept in balance [37].

For the SAUR NJ tree, there is an evident expansion of the *I. batatas SAUR* gene family, and these sequence numbers are similar to those in other plants that have undergone expansion, such as *Medicago truncatula* (141 *SAUR* genes) and soybean (236 *SAUR* genes) [25]. Further studies are required to determine whether the large expansion of *SAUR* genes retains the same functions as their *A. thaliana* homologs.

### Auxin signalling genes have multiple roles in sweet potato growth and development

The diversity of CREs in the promoter regions of the *ARF* genes indicates that miscellaneous transcriptional cues can cause different family members to have varying effects. There were several regulatory elements involved in hormone signalling. Auxin signalling genes also have roles in stress responses [38, 39] and plant development, and the presence of the corresponding regulatory elements in the promoter sequences may help mediate these responses. These responses were confirmed by the up-regulation of some of the auxin signalling genes in response to hormone and stress treatments (Figs. S9 and S10).

The SAUR protein family has not been functionally characterized in *I. batatas* to date, but the functions of SAUR proteins in *A. thaliana* are characterized. The function of *Arabidopsis* genes can be categorized by their membership in the same clade or belonging to the same subcellular compartment [25, 40]. For example, *IbSAUR61* and *IbSAUR64* (up-regulated) are part of Clade IX, whose *Arabidopsis* members promote plant growth by mediating ethylene signalling [25]. Thus, the up-regulated expression of *IbSAUR61* at 30 DAT in SR and not FR (Fig. S8b) suggests a role in tuberization via an auxin-ethylene control of root development [41]. *IbSAUR10* and *IbSAUR34* (down-regulated) belong to Clade IV, of which AtSAUR41 can be induced by ABA to elicit several functions which include calcium homeostasis, salt tolerance, and cell expansion. *IbSAUR13* (up-regulated) and *IbSAUR41* (not a DEG) belong to Clade I, of which the *A. thaliana* members promote cell expansion via activation of the plasma membrane H ^+^ -ATPase [25]. Transcription of Clade I genes is regulated by a complex that integrates signals from auxin, gibberellin (GA), brassinosteroids (BR), JA, light, shade, and heat [25]. The *SAUR* DEGs from this study should be investigated to understand how they integrate multiple signalling pathways.

### Auxin signalling genes are important for tuberization

The differential expression analysis results were similar to those reported by Rüscher et al. [42] who compared the transcriptomes of initiating cassava SRs and cassava non-bulked roots. Several *ARF*, *Aux*/*IAA*, and *SAUR* genes were up-regulated, while a *GH3* gene was down-regulated. Also, the agreement of the in silico and qRT-PCR expression results highlights that the gene expression pattern for these genes are not cultivar-specific.

The expression analysis supported the reports that ARFs participate in tuberization. The *S. tuberosum* homolog of IbARF8, i.e., StARF8, is a potato tuberization marker that is regulated by StBEL5-POTH1 (BEL5-LIKE HOMEODOMAIN—POTATO HOMEOBOX 1) [43]. Pratt and Zhang [19] found that *ItfARF8* was expressed highly in root tissue and is likely to have important roles during tuberization. It is worth noting that *IbARF8*, along with other auxin signalling genes that were up-regulated during SR formation, were expressed strongly in green stems and/or leaves. This observation may be because auxin is transported on a gradient from the shoot tip to the root, so that the higher auxin concentrations in the green plant parts may have elicited the higher gene expression in these parts. However, this does not preclude the utility of studying these genes in future experiments, since *ARF8* homologs are important regulators of tuberization in other crops.

Some of the *IbARF* genes did not show differential expression between FRs and are probably involved in one or more of the other diverse roles ARFs have in plants [5]. For example, *IbARF5* may be involved in drought responses and carotenoid biosynthesis [44]. In potato, *StARF5* (*MONOPTEROS*) is down-regulated in microtuber formation during non-osmotic stress, but it is up-regulated during microtuber formation in osmotic stress conditions [45]. StARF5 mediates expression of the auxin efflux carrier, StPIN1, which in turn mediates the formation of organs and vascular tissues [13]. Given that ARF5 is involved in potato microtuber induction under drought conditions [45] and is differentially expressed in cassava SRs [43], ARF5 may not be needed during optimal SR initiation conditions. Ruonala et al. [46] reviewed the multiple functions of AtARF5, with one of them being xylem differentiation into metaxylem and protoxylem in the root procambial cells. In FRs, metaxylem becomes lignified, whereas in SRs there is a reduction in secondary xylem and reduced lignification. Further work is required to determine whether down-regulation of IbARF5 is linked to the suppression of lignification in SRs. Additionally, during drought stress conditions in cassava, *ARF8* is down-regulated by post-transcriptional degradation by the miRNA, *miR167* [47].

In this study, *IbARF4* and *IbARF10* were also up-regulated, and this finding agrees with that in cassava, where *MeARF4* and *MeARF10* were up-regulated in the SR cortex and parenchyma, respectively [48]. Additionally, Nie et al. [49] found that 3 *IbIAA* genes were up-regulated during sweet potato adventitious root formation, while 3 *IbIAA* genes, *ARF4* and *ARF2* were down-regulated. In potatoes (*Solanum tuberosum*), *StARF6*, was shown to be highly expressed in stolons prior to tuberization [17]. Additionally, Pratt and Zhang [19] characterized the repertoire of ARF proteins in the sweet potato relative, *Ipomoea trifida*. They found that *ItfARF16b* and *ItfARF16c* were highly expressed in the sweet potato SR compared to the FR. Further work is required to elucidate how these proteins form an integrated signalling network that allows for the fine-tuning of early auxin signalling.

This study revealed that *Aux/IAA* genes are up-regulated in *I. batatas* SR initiation, which is supported by other studies. In potato, several *Aux/IAA* RNAs (such as *StIAA14*, the homolog of the phloem-mobile *AtIAA-18/28*) were shown to be phloem-mobile and move from the shoot to the root, where they can suppress lateral root growth [50]. Several *StIAA* genes are up-regulated during the initiation of potato tuberization [18]. Additionally, *MeIAA3* was up-regulated in the parenchyma of cassava initiating and mature SRs when compared to that of FRs [48]. IbIAA1, IbIAA16 and IbIAA17 are canonical Aux/IAA proteins, so it seems that canonical auxin signalling pathways are involved in sweet potato tuberization.

IbGH3 proteins are involved in a variety of developmental responses. Based on the homology between GH3.-2, -3, -7 and Arabidopsis Group 2 GH3 proteins, the IbGH3 proteins are likely to form auxin—amino acid conjugates. The down-regulated expressions of *IbGH3.*-*1*, -*2*, -*3*, -*6*, -*7* were expected since auxin is required for the initiation of tuberization and GH3 proteins reduce the levels of bioavailable auxin. Based on homology, GH.12 and GH3.13 may be responsible for regulation of JA levels. Dong et al. [14] revealed that JA levels decline as SR formation progresses, since there is a reduction in JA biosynthetic genes at 30 DAT.

Some *SAUR* genes are implicated in SR development. For example, Ding et al. [20] found *MeSAUR12* and *MeSAUR14* to be transcriptionally up-regulated during cassava tuberization. It is likely that some *SAUR* DEGs coordinate the responses from multiple hormone signalling pathways.

### Strengths and limitations of study

Two previous studies [51, 52] analysed the microarray expression of the auxin signalling gene families. Some of their results conflicted with those of the present study since some of the contigs they analysed were labelled as *ARF*, *Aux/IAA*, *GH3* or *SAUR* genes but did not have the corresponding domains in the NCBI CDD. Therefore, our study improves upon previously reported results. Pratt and Zhang [19] identified the ARF genes in *I. trifida* and compared their expression in 35 DAT SR vs. FR. There were some similarities and differences between our results and theirs. For example, they found that *ItfARF6a* was only expressed in FR while our study indicates that the homologous *IbARF6* has relatively high expression values across all the plant parts mentioned in this study. They also found *ItfARF5* to be highly expressed in SR as opposed to our finding for *IbARF5*. This anomaly may be due to potential cultivar differences or that their primers were not validated against the sweet potato genome to ensure that the specific ARF target was being amplified. We also looked at other RNA-seq datasets for SR initiation in other sweet potato cultivars, such as Tanzania [18] and Taizhong6 [53]. The strongly expressed DEGs that were investigated in this study (|log_2_FC|> 2) were also differentially expressed in those studies, indicating that the expression of these DEGs are not cultivar-specific, and are thus good candidates for future studies on their involvement in SR initiation. Taken together, the results of our study confirm findings of previous studies and highlights crucial points for future studies on the roles of auxin signalling genes in sweet potato growth and development.

## Conclusions

This study identified 29 *ARF*, 39 *Aux/IAA*, 13 *GH3*, and 200 *SAUR* genes in sweet potato through a genome-wide analysis. These gene families are evolutionarily conserved with similar domain structure and organization. The expression patterns in tuberizing and fibrous roots suggested the potential involvement of some of these genes during tuber initiation based on their differential expression patterns. Some of these genes include: *IbARF12* (which encodes a transcriptional activator), *IbIAA17* (which encodes a canonical Aux/IAA protein), *IbGH3.5* (which encodes a protein that reduces bioavailable auxin levels), *IbSAUR-3, -4* (possibly involved in regulating ABA levels) and *IbSAUR61* (possibly involved in ethylene signalling). The PPI network generated by STRINGdb also suggests that the proteins encoded by these genes form a complex regulatory network. These results contribute to a greater understanding of the diversity of auxin signalling genes in sweet potato and their putative roles in tuberization. These results are important for further studies on the involvement of auxin during tuberization and contributes additional evidence that will improve future genome annotations for this crop.

Further studies are required to determine how these genes regulate the tuberization process and whether there is functional redundancy among gene family members. Analysis of loss-of-function mutants and protein–protein interaction assays can help to achieve this goal. It would also be of interest to investigate how members of these families integrate signals from other hormone signalling pathways during tuberization.

## Materials and methods

### Plant material, growing conditions, and sample collection

Whole mature root tubers (mother tubers) of sweet potato cultivar O49 were obtained from the sweet potato germplasm collection (Field Station, The University of the West Indies St. Augustine, Trinidad and Tobago). The experiment was set up in the St. Augustine district between October 2019 and November 2019. The mother tubers were planted singly in pots 5–7 cm below the soil surface with the proximal end facing downwards and left for 7 weeks. The plants were fertilized with NPK (12:24:12) fertilizer according to the manufacturer’s instructions, and they were watered as needed. The plants were checked daily to remove any pests. The plants were harvested at 49 days after transplanting (DAT) to get various tissues (fibrous roots, pencil roots, green stems, leaves, and storage roots) at various stages of development. Three biological replicates were collected per tissue investigated. All samples were processed immediately after harvesting.

### Identification of putative *ARF*, *Aux/IAA*, *GH3*, and *SAUR* genes

The sweet potato genome, protein sequences, and annotations were downloaded from the Ipomoea Genome Hub (www.sweetpotao.com). The Hidden Markov Models (HMMs) for the auxin signalling gene families (ARF [Auxin_resp (PF06507)], Aux/IAA [AUX_IAA (PF02309)], GH3 [GH3 (PF03321)], SAUR [Auxin_inducible (PF02519)]) were downloaded from Pfam (http://ftp.ebi.ac.uk/pub/databases/Pfam/current_release/). Putative auxin-responsive protein sequences were obtained using HMMER [54] to search the HMMs against the proteome on the Galaxy server (https://usegalaxy.org.au/). Predicted amino acid sequences were examined using the NCBI CDD [55] for the presence of characteristic domains. ARF sequences that lacked both B3 and aux_resp domains were eliminated. CD-HIT was used to remove redundant sequences and cluster them by percentage similarity [56, 57]. The *ARF*, *Aux/IAA*, and *GH3* sequences were named according to their homology to the *Arabidopsis* homologs, followed by chromosome location. The *SAUR* genes were named based on ascending chromosome location, as was done in previous studies [58, 59].

This version of the sweet potato genome does not have the coordinates of transcript isoforms, so any novel isoforms from the RNA-seq alignments were manually curated. Briefly, Stringtie was used to identify novel alignments that were not present in the genome annotation [60]. StringtieMerge was used to merge all the alignments from the transcriptome data coming from the different sweet potato samples. GffCompare [61] was used to compare the StringtieMerge alignments with that of the reference annotation to detect novel isoforms. These isoforms were visualized in Trackster on the usegalaxy.eu platform.

### Prediction of protein properties, motif analysis and phylogenetic construction

The molecular weight and pI of the ARF, Aux/IAA, and GH3 proteins were predicted using the online tools on the ExPASy website (https://web.expasy.org/protparam/) while that of the SAUR proteins were predicted using http://www.bioinformatics.org/sms2/protein_mw.html and http://www.bioinformatics.org/sms2/protein_iep.html. Plantm-Ploc was used to predict the subcellular location of the predicted ARF and Aux/IAA proteins [62]. CELLO2GO was used to predict the GH3 and SAUR protein locations [63]. The intron–exon structures of the genes were visualized using the Gene Structure Display Server [64]. MEME v.5.4.1 [65] was used to investigate the presence of conserved motifs within the protein sequences. Certain motifs were further investigated using InterProScan (https://www.ebi.ac.uk/interpro/search/sequence-search) and/ or SMART [66]. The TBTools program [67] was used to visualize some of the motifs.

The *S. tuberosum* ARF and Aux/IAA protein sequences were downloaded from the studies performed by Song et al. [13] and Gao et al. [18] respectively; *A. thaliana* sequences were downloaded from UniProtKB [68], and *Ipomoea triloba* and *S. tuberosum* GH3 sequences were obtained from Ensembl Plants (https://plants.ensembl.org/index.html). The sequences were aligned using the ClustalW program [69] in the BioEdit software. The aligned sequences were used to construct a neighbour-joining phylogenetic tree in MEGA 11 [70] with 1000 bootstrap replicates and default parameters.

### Analysis of *cis*-acting regulatory elements in the promoter sequences

The Plant CARE database [71] was used to identify the putative *cis*-acting regulatory elements in the promoter regions of the auxin signalling genes. The 2 kb genomic DNA sequences upstream of the predicted ATG initiation codon for each gene were downloaded. Only the hits that were located on the sense strand were accepted.

### In silico auxin signalling gene expression analysis

Three sets of publicly available RNA-seq datasets were downloaded; SRX4715098-SRX4715137 and PRJNA511028 from NCBI SRA [16] and all PRJCA000640 BioProject accessions [26] obtained from the National Genomics Data Center (NGDC) (https://ngdc.cncb.ac.cn/). All the Bioinformatics analyses below were conducted on the Galaxy server (https://usegalaxy.org). The SRX4715098-SRX4715137 samples consisted of sweet potato cv. Beauregard (with four biological replicates each) at four time points: 10 DAT (days after transplanting) undifferentiated root, 20 DAT undifferentiated root, 30 DAT FR, 30 DAT SR, 40 DAT FR, 40 DAT SR, 50 DAT FR, and 50 DAT SR. The PRJNA511028 samples consisted of FR, leaf, and stem samples under ABA, MeJA, SA, drought, salt, and cold treatments and the corresponding controls. The NGDC reads consisted of one biological replicate of various sweet potato plant parts (shoot, young leaf, mature leaf, stem, FR, initiating tuberous root (ITR), expanding tuberous root (ETR), mature tuberous root (MTR)) for two cultivars (Xuzi3 and Yan252). After ensuring that the reads passed FASTQC quality control [72], the reads were mapped to the reference genome with STAR [73], and the reads were quantified with featureCounts [74]. The featureCounts outputs for the NCBI datasets were used as input for DESeq2 [75] to obtain differentially expressed genes (DEGs). The following comparisons were made for differential gene expression in the SRX4715098-SRX4715137 datasets: 20 DAT vs. 10 DAT; 30 DAT SR vs. 30 DAT FR; 40 DAT SR vs. 40 DAT FR and 50 DAT SR vs. 50 DAT FR. DEGs were those with an FDR (false discovery rate) of ≤ 0.05 and |log_2_(fold change)|≥ 1. DEGS were filtered from the PRJNA511028 samples with a FDR of ≤ 0.05 and |log_2_(fold change)|≥ 2. The featureCounts outputs for the NGDC datasets were converted to FPKM values using FPKM Count from the RseQC package [76]. The expression heatmaps were generated in Galaxy or TBTools [67].

### qRT-PCR analysis of gene expression

RNA was isolated from the various tissues using the method described by Gromadka et al. [77], except that standard acidic phenol chloroform extraction was used instead, as described by Bowrin et al. [78]. Genomic DNA was removed from the samples with the TURBO DNA-*free*™ DNase treatment kit (Invitrogen, Carlsbad, CA, United States) as per the manufacturer’s instructions. The A_260_/A_280_ and A_260_/A_230_ values were measured on a NanoDrop 2000 spectrophotometer (Thermo Fisher Scientific, Waltham, MA, United States). RNA integrity was checked via agarose gel electrophoresis.

First-strand cDNA synthesis was performed with 1 μg of RNA per reaction using the Superscript IV Reverse Transcriptase kit (Invitrogen, Carlsbad, CA, United States) as per the manufacturer’s directions. The cDNA was diluted in a 1:1 ratio with sterile water (Sigma-Aldrich, Burlington, Massachusetts, United States) and 1 μL of diluted cDNA was used per reaction. The primers for the reactions are listed in Table S1. The primers for the housekeeping gene (*COX*) were taken from the study by Park et al. [79]. No template controls were also included, to ensure that samples did not have exogenous nucleic acid contamination.

The cDNA was obtained from three pooled biological replicates of tissue. The primers were used in qRT-PCR reactions each containing: 25 μL Power SYBR® Green Master Mix (Invitrogen), 200 nM forward primer, 200 nM reverse primer, 1 μL of cDNA and 23 μL of sterile nuclease-free water (Sigma-Aldrich) with a final reaction volume of 50 μL. Each reaction had three technical replicates and was run on a qTower3 thermal cycler (Analytik Jena, Jena, Germany) with the following cycling parameters: 10 min initial denaturation followed by 40 cycles of 15 s at 95 ℃ and 60 s at 55 ℃. Melting curve analysis was conducted after (for 6 s in the range of 60 ℃ to 95 ℃) and the results were analysed using the qPCRSoft program (Analytic Jena, Jena, Germany). PCR efficiencies were determined from the raw amplification curve data using Real Time PCR Miner [80]. With this method, the calculated PCR efficiency of 2 corresponds to 100%. The Pfaffl method was used to normalize the expression levels to the housekeeping gene (*COX*) expression levels [81]. Statistical analyses (Kruskal–Wallis H test or Student’s *t*-test (*p* < 0.05)) were conducted with IBM SPSS Statistics version 28.

### Construction of Predicted Protein–Protein Interaction (PPI) network

To further understand the roles of the DEGs in tuber initiation, a PPI network was constructed. Interaction networks of DEGs from the same gene family were examined, to distinguish their roles during tuberization. The protein sequences for the DEGs were used as queries in the STRING database to obtain the PPI networks, based on their *A. thaliana* homologs [82].

### Supplementary Information

**Additional file 1:** **Fig S1.** Exon-intron structure of IbAux/IAA genes (figure created on the GSDS server). The left panel illustrates a neighbour-joining (NJ) phylogenetic tree based on the aligned sequences with 1000 bootstrap replicates. Sequences with similar intron-exon structure cluster together in the NJ tree.

**Additional file 2:** **Fig S2.** Exon-intron structure of IbGH3genes (figure created on the GSDS server). The left panel illustrates a neighbour-joining (NJ) phylogenetic tree based on the aligned sequences with 1000 bootstrap replicates. Sequences with similar intron-exon structure cluster together in the NJ tree.

**Additional file 3:** **Fig S3.** Exon-intron structure of IbSAUR genes (figure created on the GSDS server). The left panel illustrates a neighbour-joining (NJ) phylogenetic tree based on the aligned sequences with 1000 bootstrap replicates. Sequences with similar intron-exon structure cluster together in the NJ tree.

**Additional file 4:** **Fig S4.** Motifs detected in IbARF sequences with MEME. The coloured rectangles represent the 20 unique motifs that were found in the sequences, with the consensus motif sequences shown in the bottom panel.

**Additional file 5:** **Fig S5.** Motifs detected in IbGH3 sequences with MEME. The coloured rectangles represent the 20 unique motifs that were found in the sequences.

**Additional file 6:** **Fig S6.** Motifs detected in IbSAUR sequences with MEME. The coloured rectangles represent different motifs, with the 10 unique motif sequence logos.

**Additional file 7:** **Fig S7.** Neighbour-joining phylogenetic trees showing the phylogenetic relationships between: a ARF, b Aux/IAA c GH3 and d SAUR sequences. The trees were constructed using 1000 bootstrap replicates in MEGA 11. The I. batatas sequences were represented by a red circle. The numbers on each branch represent the percentage of replicate trees that clustered together in the bootstrap test.

**Additional file 8:** **Fig S8.** Heatmap showing the gene expression of IbSAUR genes obtained from: a RNA-seq data [26] obtained from various tissues for both Xuzi3 and Yan252 sweet potato cultivars. The colour scale bar represents the log_2_(FPKM + 1) values. b RNA-seq data [16] obtained from FRs and SRs at various stages of development. The colour scale bar shows that blue indicates down-regulated expression, red represents up-regulated expression and grey boxes indicate no expression. Colours represent log_2_FC. The raw log_2_FC data is indicated for statistically significant (adj. *p*-val. < 0.05) differential expression only.

**Additional file 9:** **Fig S9.** Heatmap showing the expression of *IbARF*, *IbIAA*, and *IbGH3* genes obtained from publicly available RNA-seq data (PRJNA511028). The transcriptomes were sequenced from fibrous roots (FR), leaf, and stem of sweet potato cultivar Xushu18 under ABA, MeJA, SA, drought, salt, and cold treatments relative to a control (CK). The colour scale bar represents the log_2_FC values, with blue indicating down-regulation, red indicating up-regulation and grey boxes indicating no expression. Statistically significant fold changes (adj. *p*-val. < 0.05 and |log_2_FC ≥ 2|) are represented by an asterisk.

**Additional file 10:** **Fig S10.** Heatmap showing the expression of *IbSAUR* genes obtained from publicly available RNA-seq data (PRJNA511028). The transcriptomes were sequenced from fibrous roots (FR), leaf, and stem of sweet potato cultivar Xushu18 under ABA, MeJA, SA, drought, salt, and cold treatments relative to a control (CK). The colour scale bar represents the log_2_FC values, with blue indicating down-regulation, red indicating up-regulation and grey boxes indicating no expression. Statistically significant fold changes (adj. *p*-val. < 0.05 and |log_2_FC ≥ 2|) are represented by an asterisk.

**Additional file 11:** **Table S1.** Primers used in qRT-PCR reactions.

**Additional file 12:** **Table S2.** Summary of I. batatas SAUR Gene Family.

**Additional file 13:** **Table S3.** FPKM values from Ding et al. [26] used for plotting heatmaps.

**Additional file 14:** **Table S4.** Cis-acting regulatory elements in the promoter regions of the auxin signalling gene families.

**Additional file 15:** **Table S5.** Cis-acting regulatory elements in the promoter regions of selected DEGs.

**Additional file 16:** **Table S6.** List of novel auxin signaling gene transcript isoforms obtained from RNA-seq alignments using StringTie (sorted by Chromosome order).

## Data Availability

The sequences used in this study were obtained from www.sweetpotao.com. The RNA-seq raw reads were obtained from the NCBI SRA (PRJNA491292 and PRJNA511028) and the NGDC (https://ngdc.cncb.ac.cn/bioproject/browse/PRJCA000640). All data generated or analysed during this study are included in this published article [and its supplementary information files].

## Abbreviations

ABA: Abscisic acid
ARF: Auxin response factor
Aux/IAA: Auxin/Indole-3-Acetic Acid
CDD: Conserved domains database
CK: Control
CRE: *cis*-Acting regulatory element
CTD: C-terminal domain
DAT: Days after transplantation
DBD: DNA-binding domain
DEG: Differentially expressed gene
ETR: Expanding tuberous root
FC: Fold change
FPKM: Fragments per kilobase per million
FR: Fibrous root
GA: Gibberellin
GH3: Gretchen-Hagen 3
GS: Green stem
ITR: Initiating tuberous root
JA: Jasmonic acid
MeJA: Methyl jasmonate
MTR: Mature tuberous root
MW: Molecular weight
NCBI: National Center for Biotechnology Information
NGDC: National Genomics Data Center
NJ: Neighbor-joining
PB1: Phox and Bem1p
pI: Isoelectric point
PPI: Protein–protein interaction
PR: Pencil root
qRT-PCR: Quantitative real-time reverse transcription polymerase chain reaction
SA: Salicylic acid
SAUR: Small auxin up-regulated RNA
SE: Standard error
SR: Storage root
SRA: Sequence Read Archive
