# Supplementary figures and images for: Genome-wide identification, characterization, and expression analysis of the sweet potato (Ipomoea batatas [L.] Lam.) ARF, Aux/IAA, GH3, and SAUR gene families
Source: BMC Plant Biol. 2023 Dec 7;23:622. doi: 10.1186/s12870-023-04598-w (PMC10701959; doi:10.1186/s12870-023-04598-w)

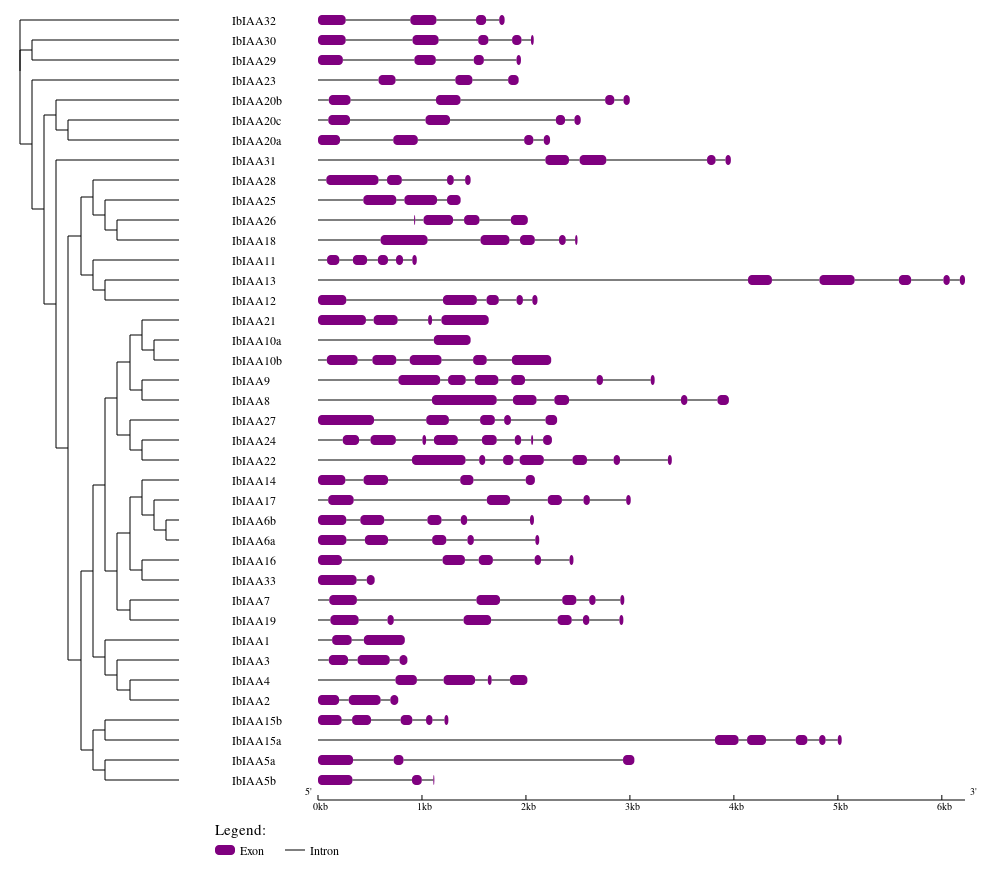

Supplement: Supplementary file 1 — Additional file 1: Fig S1. Exon-intron structure of IbAux/IAA genes (figure created on the GSDS server). The left panel illustrates a neighbour-joining (NJ) phylogenetic tree based on the aligned sequences with 1000 bootstrap replicates. Sequences with similar intron-exon structure cluster together in the NJ tree. [file 12870_2023_4598_MOESM1_ESM.png]

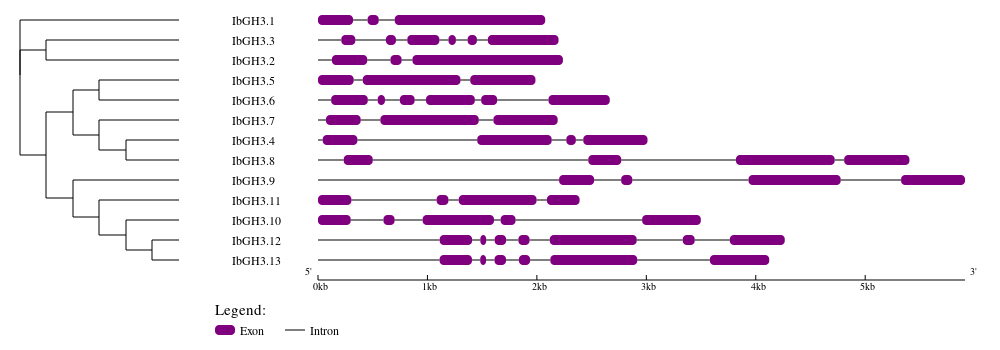

Supplement: Supplementary file 2 — Additional file 2: Fig S2. Exon-intron structure of IbGH3genes (figure created on the GSDS server). The left panel illustrates a neighbour-joining (NJ) phylogenetic tree based on the aligned sequences with 1000 bootstrap replicates. Sequences with similar intron-exon structure cluster together in the NJ tree. [file 12870_2023_4598_MOESM2_ESM.png]

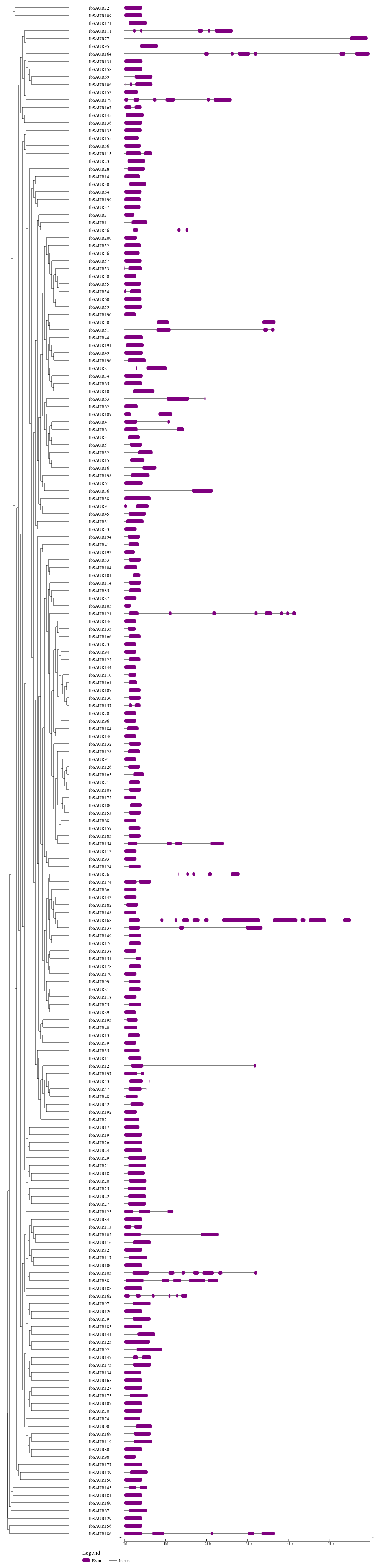

Supplement: Supplementary file 3 — Additional file 3: Fig S3. Exon-intron structure of IbSAUR genes (figure created on the GSDS server). The left panel illustrates a neighbour-joining (NJ) phylogenetic tree based on the aligned sequences with 1000 bootstrap replicates. Sequences with similar intron-exon structure cluster together in the NJ tree. [file 12870_2023_4598_MOESM3_ESM.png]

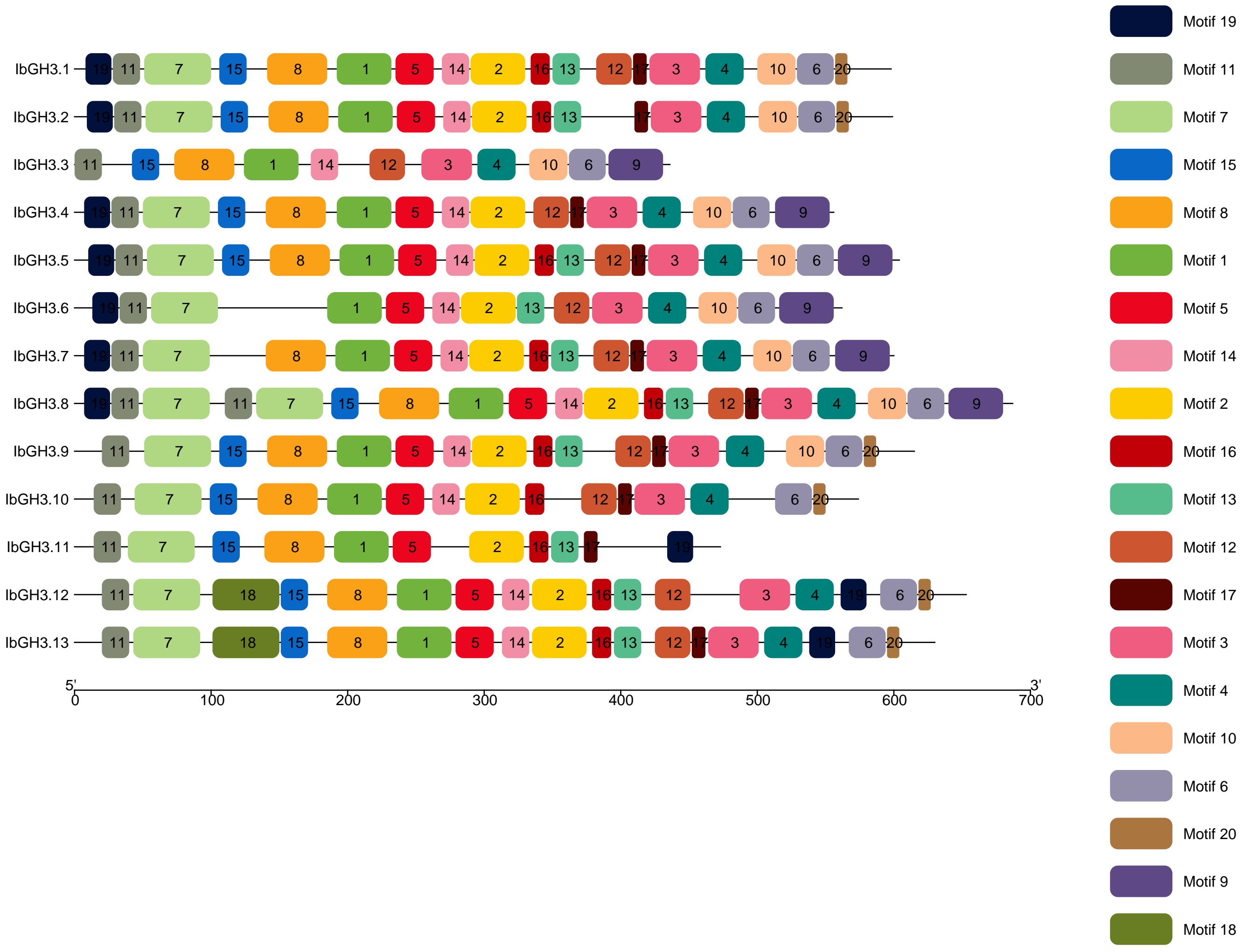

Supplement: Supplementary file 5 — Additional file 5: Fig S5. Motifs detected in IbGH3 sequences with MEME. The coloured rectangles represent the 20 unique motifs that were found in the sequences. [file 12870_2023_4598_MOESM5_ESM.pdf]

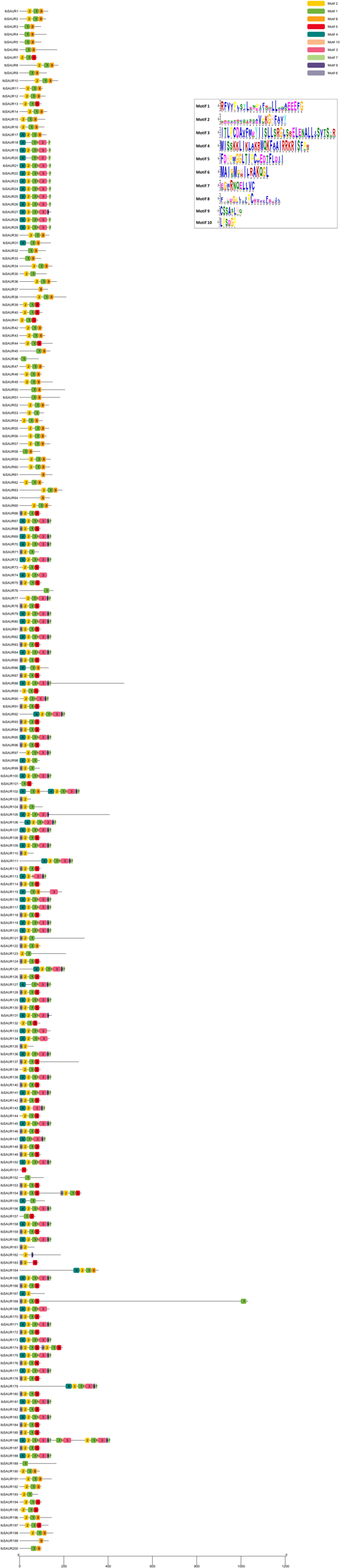

Supplement: Supplementary file 6 — Additional file 6: Fig S6. Motifs detected in IbSAUR sequences with MEME. The coloured rectangles represent different motifs, with the 10 unique motif sequence logos. [file 12870_2023_4598_MOESM6_ESM.pdf]

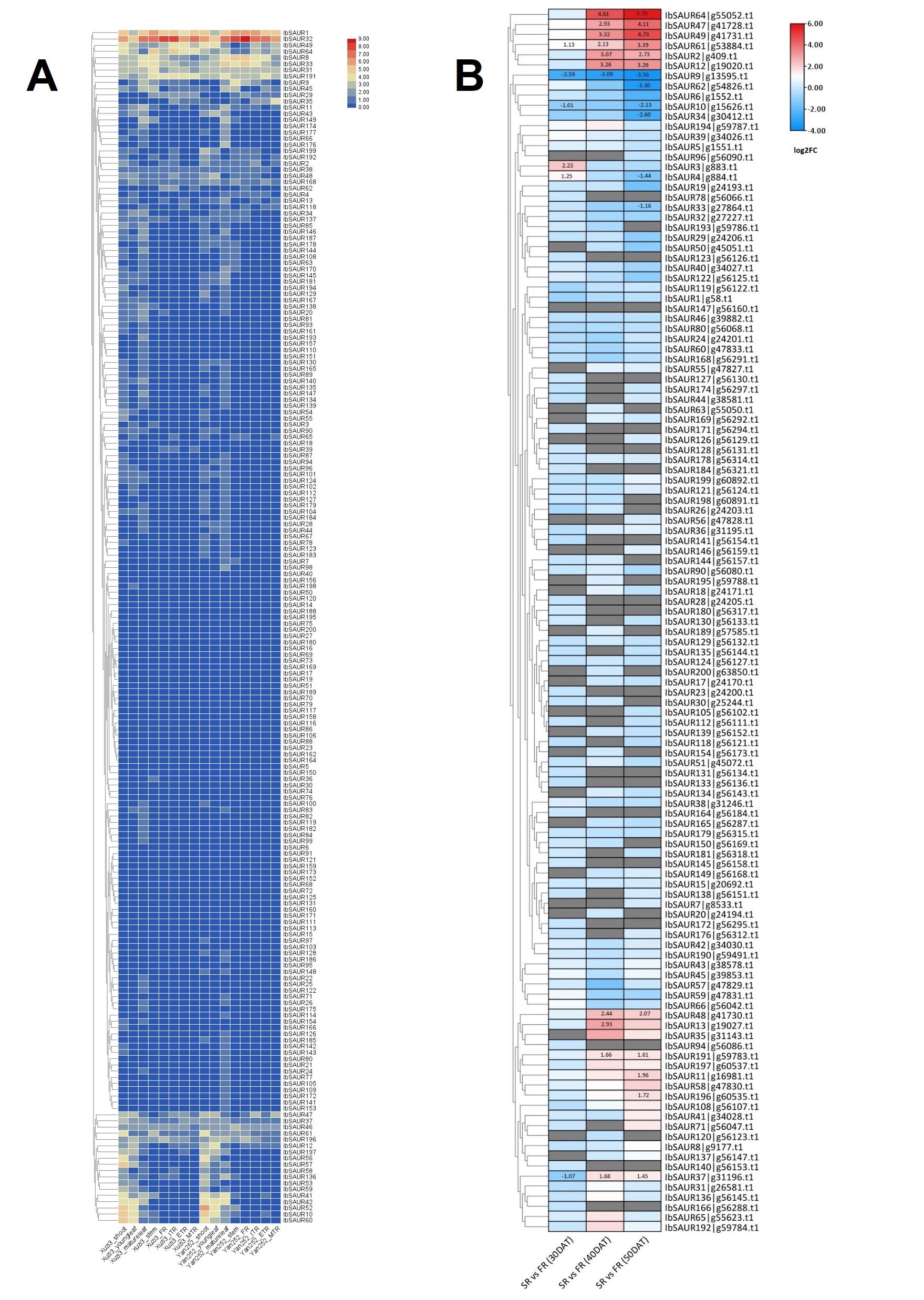

Supplement: Supplementary file 8 — Additional file 8: Fig S8. Heatmap showing the gene expression of IbSAUR genes obtained from: a RNA-seq data [26] obtained from various tissues for both Xuzi3 and Yan252 sweet potato cultivars. The colour scale bar represents the log2(FPKM + 1) values. b RNA-seq data [16] obtained from FRs and SRs at various stages of development. The colour scale bar shows that blue indicates down-regulated expression, red represents up-regulated expression and grey boxes indicate no expression. Colours represent log2FC. The raw log2FC data is indicated for statistically significant (adj. p-val. < 0.05) differential expression only. [file 12870_2023_4598_MOESM8_ESM.jpg]

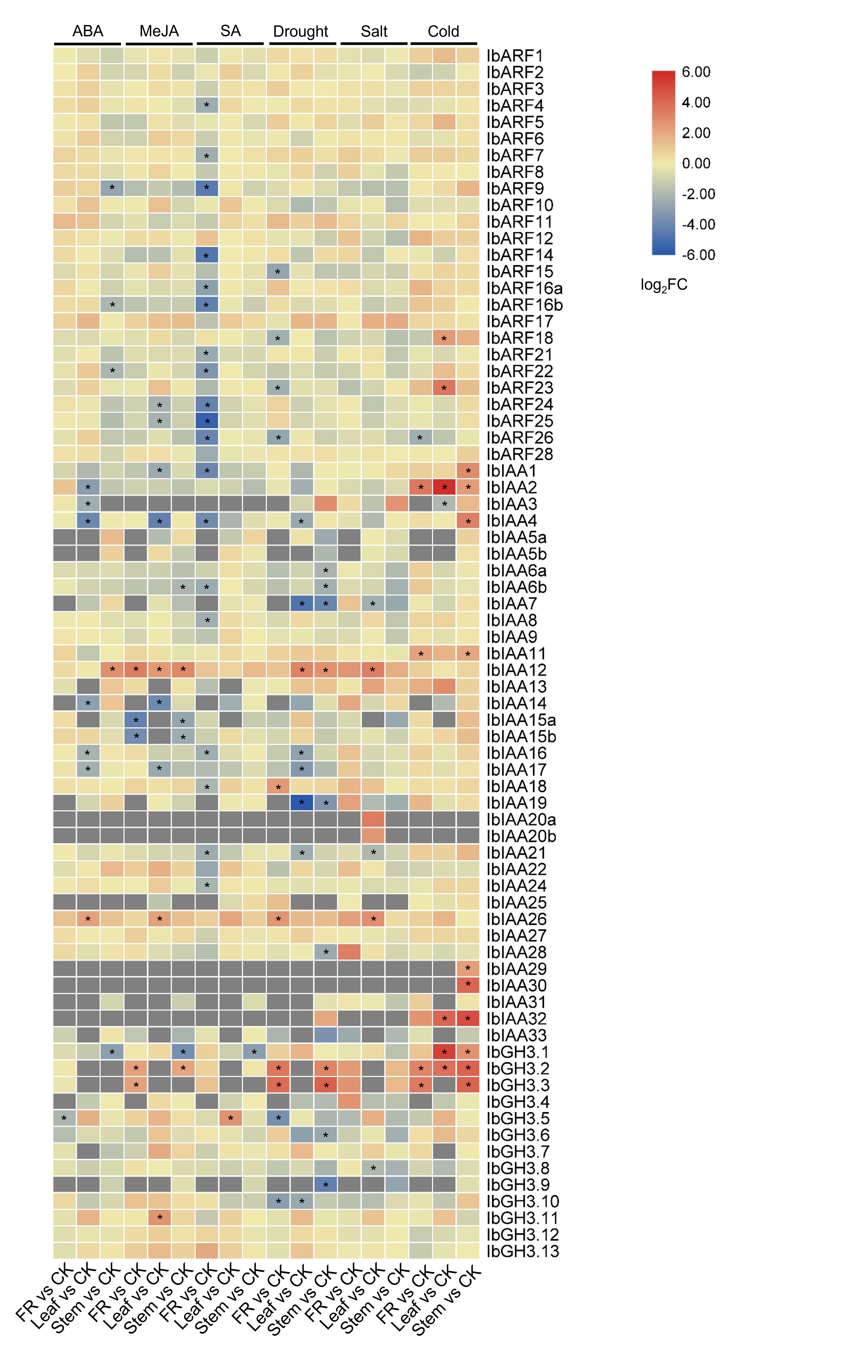

Supplement: Supplementary file 9 — Additional file 9: Fig S9. Heatmap showing the expression of IbARF, IbIAA, and IbGH3 genes obtained from publicly available RNA-seq data (PRJNA511028). The transcriptomes were sequenced from fibrous roots (FR), leaf, and stem of sweet potato cultivar Xushu18 under ABA, MeJA, SA, drought, salt, and cold treatments relative to a control (CK). The colour scale bar represents the log2FC values, with blue indicating down-regulation, red indicating up-regulation and grey boxes indicating no expression. Statistically significant fold changes (adj. p-val. < 0.05 and |log2FC ≥ 2|) are represented by an asterisk. [file 12870_2023_4598_MOESM9_ESM.png]

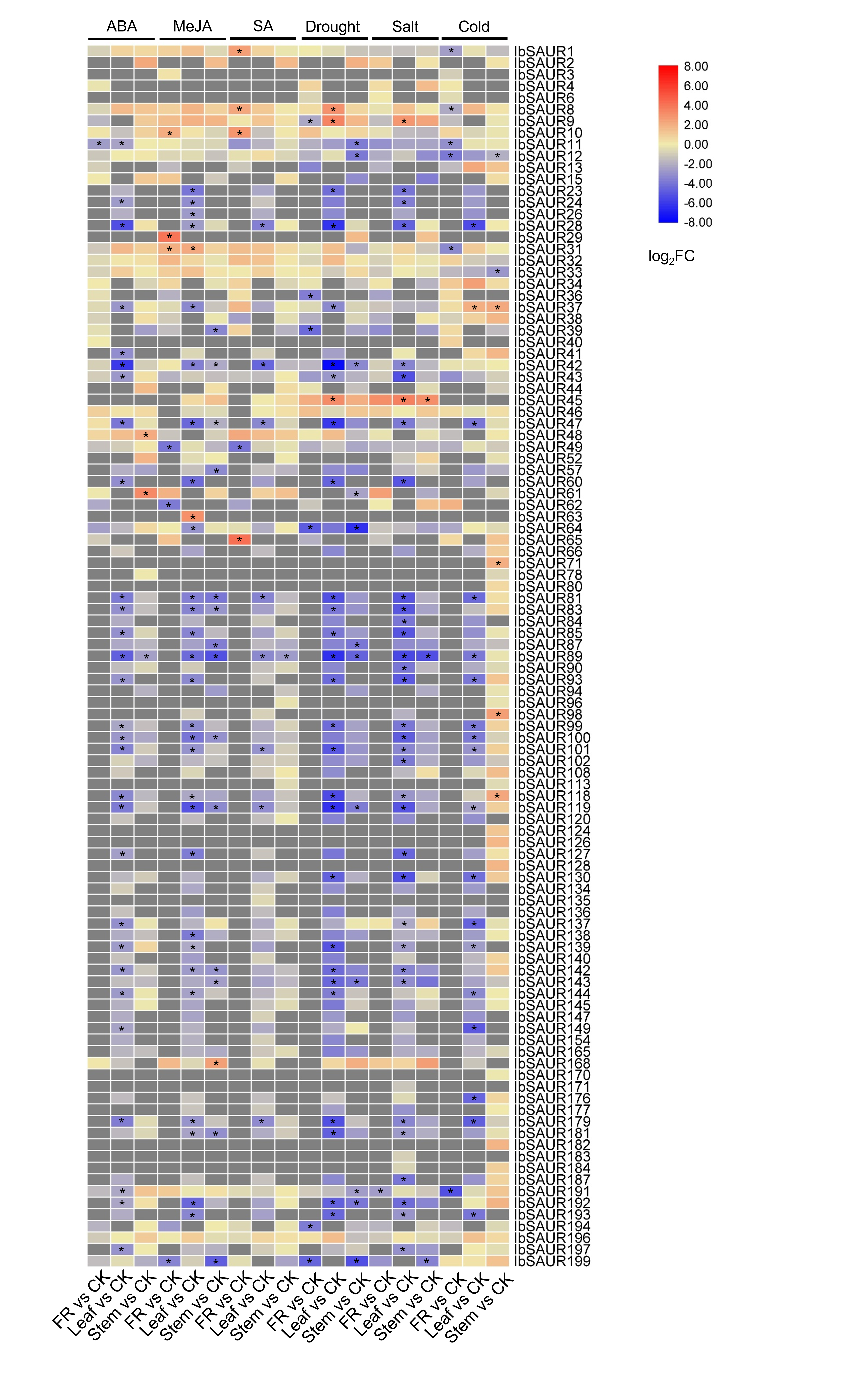

Supplement: Supplementary file 10 — Additional file 10: Fig S10. Heatmap showing the expression of IbSAUR genes obtained from publicly available RNA-seq data (PRJNA511028). The transcriptomes were sequenced from fibrous roots (FR), leaf, and stem of sweet potato cultivar Xushu18 under ABA, MeJA, SA, drought, salt, and cold treatments relative to a control (CK). The colour scale bar represents the log2FC values, with blue indicating down-regulation, red indicating up-regulation and grey boxes indicating no expression. Statistically significant fold changes (adj. p-val. < 0.05 and |log2FC ≥ 2|) are represented by an asterisk. [file 12870_2023_4598_MOESM10_ESM.png]
