# Supplementary material for: Genome-wide identification, characterization, and expression analysis of the sweet potato (Ipomoea batatas [L.] Lam.) ARF, Aux/IAA, GH3, and SAUR gene families
Source: BMC Plant Biol. 2023 Dec 7;23:622. doi: 10.1186/s12870-023-04598-w (PMC10701959; doi:10.1186/s12870-023-04598-w)
Supplement: Supplementary file 16 — Additional file 16: Table S6. List of novel auxin signaling gene transcript isoforms obtained from RNA-seq alignments using StringTie (sorted by Chromosome order). [file 12870_2023_4598_MOESM16_ESM.docx]

**Table S6** List of novel auxin signaling gene transcript isoforms obtained from RNA-seq alignments using StringTie (sorted by Chromosome order)

| Gene Name | Chr. | Gene ID | Transcript ID | Feature | Start | End | Width | Exon number |
| --- | --- | --- | --- | --- | --- | --- | --- | --- |
| IbARF12 | LG1 | g499 | g499.t2 | transcript | 2741537 | 2746065 | 4529 | NA |
|  |  |  |  | exon | 2741537 | 2742030 | 494 | 1 |
|  |  |  |  | exon | 2742219 | 2742409 | 191 | 2 |
|  |  |  |  | exon | 2742508 | 2743512 | 1005 | 3 |
|  |  |  |  | exon | 2743791 | 2743938 | 148 | 4 |
|  |  |  |  | exon | 2744402 | 2744477 | 76 | 5 |
|  |  |  |  | exon | 2744852 | 2744974 | 123 | 6 |
|  |  |  |  | exon | 2745051 | 2745215 | 165 | 7 |
|  |  |  |  | exon | 2745338 | 2745428 | 91 | 8 |
|  |  |  |  | exon | 2745981 | 2746065 | 85 | 9 |
|  |  |  | g499.t3 | transcript | 2742506 | 2746066 | 3561 | NA |
|  |  |  |  | exon | 2742506 | 2743512 | 1007 | 1 |
|  |  |  |  | exon | 2743791 | 2743938 | 148 | 2 |
|  |  |  |  | exon | 2744402 | 2744477 | 76 | 3 |
|  |  |  |  | exon | 2744852 | 2744974 | 123 | 4 |
|  |  |  |  | exon | 2745051 | 2745215 | 165 | 5 |
|  |  |  |  | exon | 2745338 | 2745423 | 86 | 6 |
|  |  |  |  | exon | 2745981 | 2746066 | 86 | 7 |
| IbARF26 | LG2 | g4723 | g4723.t2 | transcript | 3674821 | 3679349 | 4529 | NA |
|  |  |  |  | exon | 3674821 | 3675470 | 650 | 1 |
|  |  |  |  | exon | 3675709 | 3676880 | 1172 | 2 |
|  |  |  |  | exon | 3677283 | 3678166 | 884 | 3 |
|  |  |  |  | exon | 3678298 | 3678360 | 63 | 4 |
|  |  |  |  | exon | 3678904 | 3679349 | 446 | 5 |
| IbARF4 | LG2 | g4753 | g4753.t2 | transcript | 3851819 | 3855583 | 3765 | NA |
|  |  |  |  | exon | 3851819 | 3852694 | 876 | 1 |
|  |  |  |  | exon | 3853083 | 3853139 | 57 | 2 |
|  |  |  |  | exon | 3853382 | 3853579 | 198 | 3 |
|  |  |  |  | exon | 3853686 | 3853770 | 85 | 4 |
|  |  |  |  | exon | 3853902 | 3853991 | 90 | 5 |
|  |  |  |  | exon | 3854151 | 3854315 | 165 | 6 |
|  |  |  |  | exon | 3854867 | 3854986 | 120 | 7 |
|  |  |  |  | exon | 3855069 | 3855144 | 76 | 8 |
|  |  |  |  | exon | 3855421 | 3855583 | 163 | 9 |
| IbARF2 | LG2 | g8727 | g8727.t2 | transcript | 33248143 | 33251933 | 3791 | NA |
|  |  |  |  | exon | 33248143 | 33248715 | 573 | 1 |
|  |  |  |  | exon | 33248833 | 33250068 | 1236 | 2 |
|  |  |  |  | exon | 33250408 | 33251303 | 896 | 3 |
|  |  |  |  | exon | 33251382 | 33251933 | 552 | 4 |
| IbIAA11 | LG3 | g9720 | g9720.t2 | transcript | 1980396 | 1982687 | 2292 | NA |
|  |  |  |  | exon | 1980396 | 1980844 | 449 | 1 |
|  |  |  |  | exon | 1981740 | 1982074 | 335 | 2 |
|  |  |  |  | exon | 1982206 | 1982337 | 132 | 3 |
|  |  |  |  | exon | 1982619 | 1982687 | 69 | 4 |
|  |  |  | g9720.t3 | transcript | 1981935 | 1983222 | 1288 | NA |
|  |  |  |  | exon | 1981935 | 1982074 | 140 | 1 |
|  |  |  |  | exon | 1982206 | 1982341 | 136 | 2 |
|  |  |  |  | exon | 1982620 | 1982687 | 68 | 3 |
|  |  |  |  | exon | 1982777 | 1983222 | 446 | 4 |
| IbIAA4 | LG4 | g13218 | g13218.t2 | transcript | 3047227 | 3049178 | 1952 | NA |
|  |  |  |  | exon | 3047227 | 3047632 | 406 | 1 |
|  |  |  |  | exon | 3047890 | 3048192 | 303 | 2 |
|  |  |  |  | exon | 3048528 | 3049178 | 651 | 3 |
| IbIAA13 | LG4 | g13071 | g13071.t2 | transcript | 2091350 | 2093795 | 2446 | NA |
|  |  |  |  | exon | 2091350 | 2091717 | 368 | 1 |
|  |  |  |  | exon | 2091814 | 2091875 | 62 | 2 |
|  |  |  |  | exon | 2092186 | 2092303 | 118 | 3 |
|  |  |  |  | exon | 2092730 | 2093064 | 335 | 4 |
|  |  |  |  | exon | 2093525 | 2093795 | 271 | 5 |
| IbIAA28 | LG6 | g21443 | g21443.t2 | transcript | 4380364 | 4382045 | 1682 | NA |
|  |  |  |  | exon | 4380364 | 4380932 | 569 | 1 |
|  |  |  |  | exon | 4381015 | 4381156 | 142 | 2 |
|  |  |  |  | exon | 4381592 | 4381656 | 65 | 3 |
|  |  |  |  | exon | 4381762 | 4382045 | 284 | 4 |
| IbIAA8 | LG7 | g29122 | g29122.t2 | transcript | 28177795 | 28182120 | 4326 | NA |
|  |  |  |  | exon | 28177795 | 28178242 | 448 | 1 |
|  |  |  |  | exon | 28178525 | 28178586 | 62 | 2 |
|  |  |  |  | exon | 28179663 | 28179804 | 142 | 3 |
|  |  |  |  | exon | 28179976 | 28180202 | 227 | 4 |
|  |  |  |  | exon | 28180359 | 28181012 | 654 | 5 |
|  |  |  |  | exon | 28181859 | 28182120 | 262 | 6 |
|  |  |  | g29122.t3 | transcript | 28177884 | 28182120 | 4237 | NA |
|  |  |  |  | exon | 28177884 | 28178242 | 359 | 1 |
|  |  |  |  | exon | 28178525 | 28178586 | 62 | 2 |
|  |  |  |  | exon | 28179663 | 28179804 | 142 | 3 |
|  |  |  |  | exon | 28179976 | 28180202 | 227 | 4 |
|  |  |  |  | exon | 28180359 | 28181015 | 657 | 5 |
|  |  |  |  | exon | 28181859 | 28182120 | 262 | 6 |
| IbARF8 | LG7 | g29590 | g29590.t2 | transcript | 31358459 | 31362708 | 4250 | NA |
|  |  |  |  | exon | 31358459 | 31358518 | 60 | 1 |
|  |  |  |  | exon | 31358739 | 31358814 | 76 | 2 |
|  |  |  |  | exon | 31360844 | 31360928 | 85 | 3 |
|  |  |  |  | exon | 31361018 | 31361173 | 156 | 4 |
|  |  |  |  | exon | 31361278 | 31361334 | 57 | 5 |
|  |  |  |  | exon | 31361435 | 31361533 | 99 | 6 |
|  |  |  |  | exon | 31361661 | 31361773 | 113 | 7 |
|  |  |  |  | exon | 31362002 | 31362708 | 707 | 8 |
|  |  |  | g29590.t3 | transcript | 31359889 | 31362708 | 2820 | NA |
|  |  |  |  | exon | 31359889 | 31359979 | 91 | 1 |
|  |  |  |  | exon | 31360844 | 31360928 | 85 | 2 |
|  |  |  |  | exon | 31361018 | 31361173 | 156 | 3 |
|  |  |  |  | exon | 31361278 | 31361334 | 57 | 4 |
|  |  |  |  | exon | 31361435 | 31361533 | 99 | 5 |
|  |  |  |  | exon | 31361661 | 31361773 | 113 | 6 |
|  |  |  |  | exon | 31362002 | 31362708 | 707 | 7 |
| IbARF16b | LG8 | g31598 | g31598.t2 | transcript | 8143716 | 8146335 | 2620 | NA |
|  |  |  |  | exon | 8143716 | 8144075 | 360 | 1 |
|  |  |  |  | exon | 8144201 | 8144316 | 116 | 2 |
|  |  |  |  | exon | 8144525 | 8144620 | 96 | 3 |
|  |  |  |  | exon | 8144761 | 8144817 | 57 | 4 |
|  |  |  |  | exon | 8144907 | 8145062 | 156 | 5 |
|  |  |  |  | exon | 8145169 | 8145253 | 85 | 6 |
|  |  |  |  | exon | 8145603 | 8145693 | 91 | 7 |
|  |  |  |  | exon | 8145789 | 8145953 | 165 | 8 |
|  |  |  |  | exon | 8146056 | 8146175 | 120 | 9 |
|  |  |  |  | exon | 8146260 | 8146335 | 76 | 10 |
|  |  |  | g31598.t3 | transcript | 8144266 | 8145953 | 1688 | NA |
|  |  |  |  | exon | 8144266 | 8144316 | 51 | 1 |
|  |  |  |  | exon | 8144525 | 8144625 | 101 | 2 |
|  |  |  |  | exon | 8144761 | 8144817 | 57 | 3 |
|  |  |  |  | exon | 8144907 | 8145062 | 156 | 4 |
|  |  |  |  | exon | 8145169 | 8145253 | 85 | 5 |
|  |  |  |  | exon | 8145603 | 8145693 | 91 | 6 |
|  |  |  |  | exon | 8145789 | 8145953 | 165 | 7 |
| IbARF16a | LG8 | g31604 | g31604.t2 | transcript | 8144266 | 8145953 | 1688 | NA |
|  |  |  |  | exon | 8144266 | 8144316 | 51 | 1 |
|  |  |  |  | exon | 8144525 | 8144625 | 101 | 2 |
|  |  |  |  | exon | 8144761 | 8144817 | 57 | 3 |
|  |  |  |  | exon | 8144907 | 8145062 | 156 | 4 |
|  |  |  |  | exon | 8145169 | 8145253 | 85 | 5 |
|  |  |  |  | exon | 8145603 | 8145693 | 91 | 6 |
|  |  |  |  | exon | 8145789 | 8145953 | 165 | 7 |
| IbARF17 | LG8 | g32239 | g32239.t2 | transcript | 13518649 | 13520755 | 2107 | NA |
|  |  |  |  | exon | 13518649 | 13519147 | 499 | 1 |
|  |  |  |  | exon | 13519260 | 13519360 | 101 | 2 |
|  |  |  |  | exon | 13519825 | 13519940 | 116 | 3 |
|  |  |  |  | exon | 13520058 | 13520152 | 95 | 4 |
|  |  |  |  | exon | 13520232 | 13520297 | 66 | 5 |
|  |  |  |  | exon | 13520420 | 13520572 | 153 | 6 |
|  |  |  |  | exon | 13520669 | 13520755 | 87 | 7 |
|  |  |  | g32239.t3 | transcript | 13518662 | 13523183 | 4522 | NA |
|  |  |  |  | exon | 13518662 | 13519360 | 699 | 1 |
|  |  |  |  | exon | 13519825 | 13519940 | 116 | 2 |
|  |  |  |  | exon | 13520058 | 13520153 | 96 | 3 |
|  |  |  |  | exon | 13520241 | 13520297 | 57 | 4 |
|  |  |  |  | exon | 13520420 | 13520572 | 153 | 5 |
|  |  |  |  | exon | 13520669 | 13520753 | 85 | 6 |
|  |  |  |  | exon | 13521065 | 13521155 | 91 | 7 |
|  |  |  |  | exon | 13521297 | 13521462 | 166 | 8 |
|  |  |  |  | exon | 13521604 | 13521717 | 114 | 9 |
|  |  |  |  | exon | 13521802 | 13521874 | 73 | 10 |
|  |  |  |  | exon | 13521956 | 13522103 | 148 | 11 |
|  |  |  |  | exon | 13522176 | 13522750 | 575 | 12 |
|  |  |  |  | exon | 13523050 | 13523183 | 134 | 13 |
|  |  |  | g32239.t4 | transcript | 13518666 | 13520755 | 2090 | NA |
|  |  |  |  | exon | 13518666 | 13519147 | 482 | 1 |
|  |  |  |  | exon | 13519260 | 13519360 | 101 | 2 |
|  |  |  |  | exon | 13519825 | 13519940 | 116 | 3 |
|  |  |  |  | exon | 13520058 | 13520153 | 96 | 4 |
|  |  |  |  | exon | 13520241 | 13520297 | 57 | 5 |
|  |  |  |  | exon | 13520420 | 13520572 | 153 | 6 |
|  |  |  |  | exon | 13520669 | 13520755 | 87 | 7 |
|  |  |  | g32239.t5 | transcript | 13519336 | 13520572 | 1237 | NA |
|  |  |  |  | exon | 13519336 | 13519360 | 25 | 1 |
|  |  |  |  | exon | 13519825 | 13519940 | 116 | 2 |
|  |  |  |  | exon | 13520058 | 13520153 | 96 | 3 |
|  |  |  |  | exon | 13520245 | 13520297 | 53 | 4 |
|  |  |  |  | exon | 13520420 | 13520572 | 153 | 5 |
|  |  |  | g32239.t6 | transcript | 13519458 | 13520298 | 841 | NA |
|  |  |  |  | exon | 13519458 | 13519690 | 233 | 1 |
|  |  |  |  | exon | 13519825 | 13519940 | 116 | 2 |
|  |  |  |  | exon | 13520058 | 13520153 | 96 | 3 |
|  |  |  |  | exon | 13520241 | 13520298 | 58 | 4 |
|  |  |  | g32239.t7 | transcript | 13519824 | 13520728 | 905 | NA |
|  |  |  |  | exon | 13519824 | 13519940 | 117 | 1 |
|  |  |  |  | exon | 13520062 | 13520153 | 92 | 2 |
|  |  |  |  | exon | 13520241 | 13520297 | 57 | 3 |
|  |  |  |  | exon | 13520420 | 13520572 | 153 | 4 |
|  |  |  |  | exon | 13520669 | 13520728 | 60 | 5 |
|  |  |  | g32239.t8 | transcript | 13521066 | 13524143 | 3078 | NA |
|  |  |  |  | exon | 13521066 | 13521155 | 90 | 1 |
|  |  |  |  | exon | 13521297 | 13521462 | 166 | 2 |
|  |  |  |  | exon | 13521604 | 13521717 | 114 | 3 |
|  |  |  |  | exon | 13521802 | 13521874 | 73 | 4 |
|  |  |  |  | exon | 13521956 | 13522103 | 148 | 5 |
|  |  |  |  | exon | 13522179 | 13522750 | 572 | 6 |
|  |  |  |  | exon | 13523050 | 13523237 | 188 | 7 |
|  |  |  |  | exon | 13523323 | 13524143 | 821 | 8 |
| IbIAA18 | LG8 | g31376 | g31376.t2 | transcript | 6690274 | 6693002 | 2729 | NA |
|  |  |  |  | exon | 6690274 | 6690513 | 240 | 1 |
|  |  |  |  | exon | 6690604 | 6690668 | 65 | 2 |
|  |  |  |  | exon | 6690902 | 6691043 | 142 | 3 |
|  |  |  |  | exon | 6691146 | 6691423 | 278 | 4 |
|  |  |  |  | exon | 6691933 | 6692444 | 512 | 5 |
|  |  |  |  | exon | 6692547 | 6693002 | 456 | 6 |
|  |  |  | g31376.t3 | transcript | 6690289 | 6692366 | 2078 | NA |
|  |  |  |  | exon | 6690289 | 6690513 | 225 | 1 |
|  |  |  |  | exon | 6690604 | 6690847 | 244 | 2 |
|  |  |  |  | exon | 6690902 | 6691043 | 142 | 3 |
|  |  |  |  | exon | 6691146 | 6691423 | 278 | 4 |
|  |  |  |  | exon | 6691933 | 6692366 | 434 | 5 |
| IbIAA31 | LG9 | g36618 | g36618.t2 | transcript | 18870097 | 18871633 | 1537 | NA |
|  |  |  |  | exon | 18870097 | 18870196 | 100 | 1 |
|  |  |  |  | exon | 18871166 | 18871633 | 468 | 2 |
| IbARF6 | LG9 | g37210 | g37210.t2 | transcript | 23722013 | 23726213 | 4201 | NA |
|  |  |  |  | exon | 23722013 | 23723114 | 1102 | 1 |
|  |  |  |  | exon | 23723262 | 23723374 | 113 | 2 |
|  |  |  |  | exon | 23723999 | 23724097 | 99 | 3 |
|  |  |  |  | exon | 23724197 | 23724253 | 57 | 4 |
|  |  |  |  | exon | 23724328 | 23724486 | 159 | 5 |
|  |  |  |  | exon | 23724582 | 23724666 | 85 | 6 |
|  |  |  |  | exon | 23724916 | 23725006 | 91 | 7 |
|  |  |  |  | exon | 23725410 | 23725574 | 165 | 8 |
|  |  |  |  | exon | 23725661 | 23725783 | 123 | 9 |
|  |  |  |  | exon | 23725888 | 23725963 | 76 | 10 |
|  |  |  |  | exon | 23726065 | 23726213 | 149 | 11 |
| IbARF9 | LG9 | g35069 | g35069.t2 | transcript | 7163460 | 7166790 | 3331 | NA |
|  |  |  |  | exon | 7163460 | 7163653 | 194 | 1 |
|  |  |  |  | exon | 7163776 | 7163929 | 154 | 2 |
|  |  |  |  | exon | 7164012 | 7164084 | 73 | 3 |
|  |  |  |  | exon | 7164165 | 7164278 | 114 | 4 |
|  |  |  |  | exon | 7164382 | 7164546 | 165 | 5 |
|  |  |  |  | exon | 7164641 | 7164731 | 91 | 6 |
|  |  |  |  | exon | 7164832 | 7164917 | 86 | 7 |
|  |  |  |  | exon | 7164999 | 7165151 | 153 | 8 |
|  |  |  |  | exon | 7165284 | 7165337 | 54 | 9 |
|  |  |  |  | exon | 7165754 | 7165849 | 96 | 10 |
|  |  |  |  | exon | 7166123 | 7166790 | 668 | 11 |
| IbSAUR46 | LG10 | g39882 | g39882.t2 | transcript | 12509748 | 12517444 | 7697 | NA |
|  |  |  |  | exon | 12509748 | 12510065 | 318 | 1 |
|  |  |  |  | exon | 12510433 | 12510505 | 73 | 2 |
|  |  |  |  | exon | 12513327 | 12513402 | 76 | 3 |
|  |  |  |  | exon | 12513528 | 12513600 | 73 | 4 |
|  |  |  |  | exon | 12513695 | 12513746 | 52 | 5 |
|  |  |  |  | exon | 12516146 | 12516207 | 62 | 6 |
|  |  |  |  | exon | 12517381 | 12517444 | 64 | 7 |
|  |  |  | g39882.t3 | transcript | 12509814 | 12517444 | 7631 | NA |
|  |  |  |  | exon | 12509814 | 12510065 | 252 | 1 |
|  |  |  |  | exon | 12510429 | 12510505 | 77 | 2 |
|  |  |  |  | exon | 12511050 | 12511156 | 107 | 3 |
|  |  |  |  | exon | 12513327 | 12513402 | 76 | 4 |
|  |  |  |  | exon | 12513528 | 12513600 | 73 | 5 |
|  |  |  |  | exon | 12513695 | 12513746 | 52 | 6 |
|  |  |  |  | exon | 12516146 | 12516207 | 62 | 7 |
|  |  |  |  | exon | 12517381 | 12517444 | 64 | 8 |
|  |  |  | g39882.t4 | transcript | 12509814 | 12517444 | 7631 | NA |
|  |  |  |  | exon | 12509814 | 12510065 | 252 | 1 |
|  |  |  |  | exon | 12510429 | 12510505 | 77 | 2 |
|  |  |  |  | exon | 12511039 | 12511156 | 118 | 3 |
|  |  |  |  | exon | 12513327 | 12513402 | 76 | 4 |
|  |  |  |  | exon | 12513528 | 12513600 | 73 | 5 |
|  |  |  |  | exon | 12513695 | 12513746 | 52 | 6 |
|  |  |  |  | exon | 12516146 | 12516207 | 62 | 7 |
|  |  |  |  | exon | 12517381 | 12517444 | 64 | 8 |
| IbIAA22 | LG10 | g39034 | g39034.t2 | transcript | 6134402 | 6137431 | 3030 | NA |
|  |  |  |  | exon | 6134402 | 6134727 | 326 | 1 |
|  |  |  |  | exon | 6135188 | 6135249 | 62 | 2 |
|  |  |  |  | exon | 6135506 | 6135644 | 139 | 3 |
|  |  |  |  | exon | 6135921 | 6136153 | 233 | 4 |
|  |  |  |  | exon | 6136674 | 6137431 | 758 | 5 |
| IbARF5 | LG11 | g41733 | g41733.t2 | transcript | 2392267 | 2394536 | 2270 | NA |
|  |  |  |  | exon | 2392267 | 2392380 | 114 | 1 |
|  |  |  |  | exon | 2392830 | 2392928 | 99 | 2 |
|  |  |  |  | exon | 2393044 | 2393100 | 57 | 3 |
|  |  |  |  | exon | 2393227 | 2393379 | 153 | 4 |
|  |  |  |  | exon | 2393708 | 2393798 | 91 | 5 |
|  |  |  |  | exon | 2394023 | 2394187 | 165 | 6 |
|  |  |  |  | exon | 2394336 | 2394536 | 201 | 7 |
| IbIAA9 | LG11 | g42000 | g42000.t2 | transcript | 3988158 | 3991943 | 3786 | NA |
|  |  |  |  | exon | 3988158 | 3988626 | 469 | 1 |
|  |  |  |  | exon | 3989086 | 3989147 | 62 | 2 |
|  |  |  |  | exon | 3989836 | 3989968 | 133 | 3 |
|  |  |  |  | exon | 3990092 | 3990318 | 227 | 4 |
|  |  |  |  | exon | 3990406 | 3990575 | 170 | 5 |
|  |  |  |  | exon | 3990652 | 3991091 | 440 | 6 |
|  |  |  |  | exon | 3991535 | 3991580 | 46 | 7 |
|  |  |  |  | exon | 3991745 | 3991943 | 199 | 8 |
|  |  |  | g42000.t3 | transcript | 3988158 | 3991943 | 3786 | NA |
|  |  |  |  | exon | 3988158 | 3988626 | 469 | 1 |
|  |  |  |  | exon | 3989086 | 3989147 | 62 | 2 |
|  |  |  |  | exon | 3989836 | 3989968 | 133 | 3 |
|  |  |  |  | exon | 3990092 | 3990318 | 227 | 4 |
|  |  |  |  | exon | 3990406 | 3990575 | 170 | 5 |
|  |  |  |  | exon | 3990652 | 3991091 | 440 | 6 |
|  |  |  |  | exon | 3991745 | 3991943 | 199 | 7 |
|  |  |  | g42000.t4 | transcript | 3989835 | 3991943 | 2109 | NA |
|  |  |  |  | exon | 3989835 | 3989968 | 134 | 1 |
|  |  |  |  | exon | 3990092 | 3990321 | 230 | 2 |
|  |  |  |  | exon | 3990406 | 3990575 | 170 | 3 |
|  |  |  |  | exon | 3990652 | 3991091 | 440 | 4 |
|  |  |  |  | exon | 3991535 | 3991580 | 46 | 5 |
|  |  |  |  | exon | 3991745 | 3991943 | 199 | 6 |
|  |  |  | g42000.t5 | transcript | 3989835 | 3991943 | 2109 | NA |
|  |  |  |  | exon | 3989835 | 3989968 | 134 | 1 |
|  |  |  |  | exon | 3990092 | 3990321 | 230 | 2 |
|  |  |  |  | exon | 3990406 | 3990575 | 170 | 3 |
|  |  |  |  | exon | 3990652 | 3991091 | 440 | 4 |
|  |  |  |  | exon | 3991745 | 3991943 | 199 | 5 |
|  |  |  | g42000.t6 | transcript | 3990224 | 3991943 | 1720 | NA |
|  |  |  |  | exon | 3990224 | 3990321 | 98 | 1 |
|  |  |  |  | exon | 3990406 | 3990575 | 170 | 2 |
|  |  |  |  | exon | 3990652 | 3991079 | 428 | 3 |
|  |  |  |  | exon | 3991745 | 3991943 | 199 | 4 |
| IbARF10 | LG12 | g48029 | g48029.t2 | transcript | 8572808 | 8577750 | 4943 | NA |
|  |  |  |  | exon | 8572808 | 8572864 | 57 | 1 |
|  |  |  |  | exon | 8573404 | 8573488 | 85 | 2 |
|  |  |  |  | exon | 8573574 | 8573664 | 91 | 3 |
|  |  |  |  | exon | 8573957 | 8574121 | 165 | 4 |
|  |  |  |  | exon | 8575574 | 8575693 | 120 | 5 |
|  |  |  |  | exon | 8575787 | 8575862 | 76 | 6 |
|  |  |  |  | exon | 8576428 | 8576590 | 163 | 7 |
|  |  |  |  | exon | 8576718 | 8577750 | 1033 | 8 |
|  |  |  | g48029.t3 | transcript | 8575787 | 8578075 | 2289 | NA |
|  |  |  |  | exon | 8575787 | 8575862 | 76 | 1 |
|  |  |  |  | exon | 8576428 | 8576602 | 175 | 2 |
|  |  |  |  | exon | 8577987 | 8578075 | 89 | 3 |
| IbARF25 | LG13 | g52675 | g52675.t2 | transcript | 11072931 | 11078337 | 5407 | NA |
|  |  |  |  | exon | 11072931 | 11073223 | 293 | 1 |
|  |  |  |  | exon | 11073326 | 11073441 | 116 | 2 |
|  |  |  |  | exon | 11073653 | 11073748 | 96 | 3 |
|  |  |  |  | exon | 11073908 | 11073964 | 57 | 4 |
|  |  |  |  | exon | 11074058 | 11074213 | 156 | 5 |
|  |  |  |  | exon | 11074310 | 11074394 | 85 | 6 |
|  |  |  |  | exon | 11074738 | 11074828 | 91 | 7 |
|  |  |  |  | exon | 11074923 | 11075087 | 165 | 8 |
|  |  |  |  | exon | 11075181 | 11075301 | 121 | 9 |
|  |  |  |  | exon | 11075410 | 11075485 | 76 | 10 |
|  |  |  |  | exon | 11075575 | 11075746 | 172 | 11 |
|  |  |  |  | exon | 11075836 | 11076776 | 941 | 12 |
|  |  |  |  | exon | 11076866 | 11077056 | 191 | 13 |
|  |  |  |  | exon | 11077161 | 11077378 | 218 | 14 |
|  |  |  |  | exon | 11077522 | 11078337 | 816 | 15 |
| IbIAA15b | LG13 | g51416 | g51416.t2 | transcript | 1909987 | 1911595 | 1609 | NA |
|  |  |  |  | exon | 1909987 | 1910265 | 279 | 1 |
|  |  |  |  | exon | 1910356 | 1910417 | 62 | 2 |
|  |  |  |  | exon | 1910550 | 1910661 | 112 | 3 |
|  |  |  |  | exon | 1910947 | 1911128 | 182 | 4 |
|  |  |  |  | exon | 1911230 | 1911595 | 366 | 5 |
| IbGH3.10 | LG13 | g54418 | g54418.t2 | transcript | 23755807 | 23761480 | 5674 | NA |
|  |  |  |  | exon | 23755807 | 23755892 | 86 | 1 |
|  |  |  |  | exon | 23755984 | 23756087 | 104 | 2 |
|  |  |  |  | exon | 23757505 | 23758205 | 701 | 3 |
|  |  |  |  | exon | 23759363 | 23759498 | 136 | 4 |
|  |  |  |  | exon | 23759560 | 23760211 | 652 | 5 |
|  |  |  |  | exon | 23760468 | 23760569 | 102 | 6 |
|  |  |  |  | exon | 23760869 | 23761480 | 612 | 7 |
| IbIAA27 | LG13 | g55286 | g55286.t2 | transcript | 29005326 | 29008079 | 2754 | NA |
|  |  |  |  | exon | 29005326 | 29005883 | 558 | 1 |
|  |  |  |  | exon | 29005935 | 29006004 | 70 | 2 |
|  |  |  |  | exon | 29006094 | 29006235 | 142 | 3 |
|  |  |  |  | exon | 29006536 | 29006753 | 218 | 4 |
|  |  |  |  | exon | 29007256 | 29008079 | 824 | 5 |
| IbGH3.12 | LG14 | g56407 | g56407.t2 | transcript | 7163211 | 7167724 | 4514 | NA |
|  |  |  |  | exon | 7163211 | 7163570 | 360 | 1 |
|  |  |  |  | exon | 7164332 | 7164633 | 302 | 2 |
|  |  |  |  | exon | 7165057 | 7165158 | 102 | 3 |
|  |  |  |  | exon | 7165344 | 7166137 | 794 | 4 |
|  |  |  |  | exon | 7166948 | 7167724 | 777 | 5 |
|  |  |  | g56407.t3 | transcript | 7164182 | 7167724 | 3543 | NA |
|  |  |  |  | exon | 7164182 | 7164633 | 452 | 1 |
|  |  |  |  | exon | 7165057 | 7165158 | 102 | 2 |
|  |  |  |  | exon | 7165344 | 7166137 | 794 | 3 |
|  |  |  |  | exon | 7166415 | 7167724 | 1310 | 4 |
| IbARF1 | LG14 | g59391 | g59391.t2 | transcript | 28394447 | 28400481 | 6035 | NA |
|  |  |  |  | exon | 28394447 | 28394774 | 328 | 1 |
|  |  |  |  | exon | 28395580 | 28395722 | 143 | 2 |
|  |  |  |  | exon | 28395876 | 28395991 | 116 | 3 |
|  |  |  |  | exon | 28396142 | 28396237 | 96 | 4 |
|  |  |  |  | exon | 28396319 | 28396375 | 57 | 5 |
|  |  |  |  | exon | 28396506 | 28396658 | 153 | 6 |
|  |  |  |  | exon | 28396744 | 28396828 | 85 | 7 |
|  |  |  |  | exon | 28397038 | 28397128 | 91 | 8 |
|  |  |  |  | exon | 28397258 | 28397422 | 165 | 9 |
|  |  |  |  | exon | 28397504 | 28397623 | 120 | 10 |
|  |  |  |  | exon | 28397739 | 28397814 | 76 | 11 |
|  |  |  |  | exon | 28398093 | 28398258 | 166 | 12 |
|  |  |  |  | exon | 28398363 | 28398823 | 461 | 13 |
|  |  |  |  | exon | 28399012 | 28399202 | 191 | 14 |
|  |  |  |  | exon | 28399527 | 28400481 | 955 | 15 |
| IbGH3.13 | LG14 | g56453 | g56453.t2 | transcript | 7568261 | 7572591 | 4331 | NA |
|  |  |  |  | exon | 7568261 | 7568984 | 724 | 1 |
|  |  |  |  | exon | 7569649 | 7570442 | 794 | 2 |
|  |  |  |  | exon | 7570628 | 7570729 | 102 | 3 |
|  |  |  |  | exon | 7571159 | 7571461 | 303 | 4 |
|  |  |  |  | exon | 7571668 | 7571730 | 63 | 5 |
|  |  |  |  | exon | 7572225 | 7572591 | 367 | 6 |
|  |  |  | g56453.t3 | transcript | 7568261 | 7572591 | 4331 | NA |
|  |  |  |  | exon | 7568261 | 7568984 | 724 | 1 |
|  |  |  |  | exon | 7569649 | 7570442 | 794 | 2 |
|  |  |  |  | exon | 7570628 | 7570729 | 102 | 3 |
|  |  |  |  | exon | 7571159 | 7571461 | 303 | 4 |
|  |  |  |  | exon | 7572225 | 7572591 | 367 | 5 |
|  |  |  | g56453.t4 | transcript | 7568279 | 7572591 | 4313 | NA |
|  |  |  |  | exon | 7568279 | 7568984 | 706 | 1 |
|  |  |  |  | exon | 7569649 | 7570442 | 794 | 2 |
|  |  |  |  | exon | 7570628 | 7570729 | 102 | 3 |
|  |  |  |  | exon | 7571159 | 7571730 | 572 | 4 |
|  |  |  |  | exon | 7572225 | 7572591 | 367 | 5 |
| IbARF28 | LG15 | g60835 | g60835.t2 | transcript | 6073407 | 6076451 | 3045 | NA |
|  |  |  |  | exon | 6073407 | 6073494 | 88 | 1 |
|  |  |  |  | exon | 6073835 | 6074201 | 367 | 2 |
|  |  |  |  | exon | 6074931 | 6076112 | 1182 | 3 |
|  |  |  |  | exon | 6076285 | 6076451 | 167 | 4 |
| IbIAA26 | LG15 | g61025 | g61025.t2 | transcript | 7370846 | 7373511 | 2666 | NA |
|  |  |  |  | exon | 7370846 | 7371176 | 331 | 1 |
|  |  |  |  | exon | 7371260 | 7371324 | 65 | 2 |
|  |  |  |  | exon | 7371627 | 7371774 | 148 | 3 |
|  |  |  |  | exon | 7371881 | 7372164 | 284 | 4 |
|  |  |  |  | exon | 7372998 | 7373511 | 514 | 5 |
|  |  |  | g61025.t3 | transcript | 7371003 | 7373511 | 2509 | NA |
|  |  |  |  | exon | 7371003 | 7371176 | 174 | 1 |
|  |  |  |  | exon | 7371260 | 7371324 | 65 | 2 |
|  |  |  |  | exon | 7371627 | 7371774 | 148 | 3 |
|  |  |  |  | exon | 7371881 | 7372176 | 296 | 4 |
|  |  |  |  | exon | 7372998 | 7373511 | 514 | 5 |
